# A New Thienopyrimidinone Chemotype Shows Multistage Activity against Plasmodium falciparum, Including Artemisinin-Resistant Parasites

**DOI:** 10.1128/Spectrum.00274-21

**Published:** 2021-09-29

**Authors:** Henriette Bosson-Vanga, Nicolas Primas, Jean-François Franetich, Catherine Lavazec, Lina Gomez, Kutub Ashraf, Maurel Tefit, Valérie Soulard, Nathalie Dereuddre-Bosquet, Roger Le Grand, Mélanie Donnette, Romain Mustière, Nadia Amanzougaghene, Shahin Tajeri, Peggy Suzanne, Aurélie Malzert-Fréon, Sylvain Rault, Patrice Vanelle, Sébastien Hutter, Anita Cohen, Georges Snounou, Pierre Roques, Nadine Azas, Prisca Lagardère, Vincent Lisowski, Nicolas Masurier, Michel Nguyen, Lucie Paloque, Françoise Benoit-Vical, Pierre Verhaeghe, Dominique Mazier

**Affiliations:** a Centre d’Immunologie et des Maladies Infectieuses (CIMI), INSERM, CNRS, Sorbonne Université, Paris, France; b Département de Parasitologie-Mycologie, UFR des Sciences Pharmaceutiques et Biologiques, Université Félix Houphouët Boigny, Abidjan, Côte d’Ivoire; c Aix Marseille Université, CNRS, ICR UMR 7273, Equipe Pharmaco-Chimie Radicalaire, Faculté de Pharmacie, Marseille, France; d Institut Cochin, Inserm U1016, CNRS UMR8104, Université de Paris, Paris, France; e Immunology of Viral Infections and Autoimmune Diseases, INSERM U1184, CEA, Université Paris Sud 11, Fontenay-aux-Roses, France; f Centre d’Etudes et de Recherche sur le Médicament de Normandie (CERMN), Normandie Université, UNICAEN, Caen, France; g Aix Marseille Université, IRD, AP-HM, SSA, VITROME, Marseille, France; h Institut des Biomolécules Max Mousseron, UMR 5247, CNRS, Université de Montpellier, ENSCM, UFR des Sciences Pharmaceutiques et Biologiques, Montpellier, France; i LCC-CNRS, Université de Toulouse, CNRS UPR 8241, UPS, Toulouse, France; j Institut de Pharmacologie et de Biologie Structurale (IPBS), Université de Toulouse, CNRS, UPS, Toulouse, France; k New Antimalarial Molecules and Pharmacological Approaches, Inserm ERL 1289, Toulouse, France; l Service Central de la Qualité et de l'Information Pharmaceutiques, APHM, Hôpital Conception, Marseille, France; m CHU de Toulouse, Service Pharmacie, Toulouse, France; University of Georgia

**Keywords:** antimalarials, 2-amino-6-phenyl-thienopyrimidin-4(3*H*)-one, multistage activity, artemisinin resistance

## Abstract

Human malaria infection begins with a one-time asymptomatic liver stage followed by a cyclic symptomatic blood stage. For decades, the research for novel antimalarials focused on the high-throughput screening of molecules that only targeted the asexual blood stages. In a search for new effective compounds presenting a triple action against erythrocytic and liver stages in addition to the ability to block the transmission of the disease *via* the mosquito vector, 2-amino-thienopyrimidinone derivatives were synthesized and tested for their antimalarial activity. One molecule, named gamhepathiopine (denoted as “M1” herein), was active at submicromolar concentrations against both erythrocytic (50% effective concentration [EC_50_] = 0.045 μM) and liver (EC_50_ = 0.45 μM) forms of Plasmodium falciparum. Furthermore, gamhepathiopine efficiently blocked the development of the sporogonic cycle in the mosquito vector by inhibiting the exflagellation step. Moreover, M1 was active against artemisinin-resistant forms (EC_50_ = 0.227 μM), especially at the quiescent stage. Nevertheless, in mice, M1 showed modest activity due to its rapid metabolization by P450 cytochromes into inactive derivatives, calling for the development of new parent compounds with improved metabolic stability and longer half-lives. These results highlight the thienopyrimidinone scaffold as a novel antiplasmodial chemotype of great interest to search for new drug candidates displaying multistage activity and an original mechanism of action with the potential to be used in combination therapies for malaria elimination in the context of artemisinin resistance.

**IMPORTANCE** This work reports a new chemical structure that (i) displays activity against the human malaria parasite Plasmodium falciparum at 3 stages of the parasitic cycle (blood stage, hepatic stage, and sexual stages), (ii) remains active against parasites that are resistant to the first-line treatment recommended by the World Health Organization (WHO) for the treatment of severe malaria (artemisinins), and (iii) reduces transmission of the parasite to the mosquito vector in a mouse model. This new molecule family could open the way to the conception of novel antimalarial drugs with an original multistage mechanism of action to fight against *Plasmodium* drug resistance and block interhuman transmission of malaria.

## INTRODUCTION

**FIG 1:**
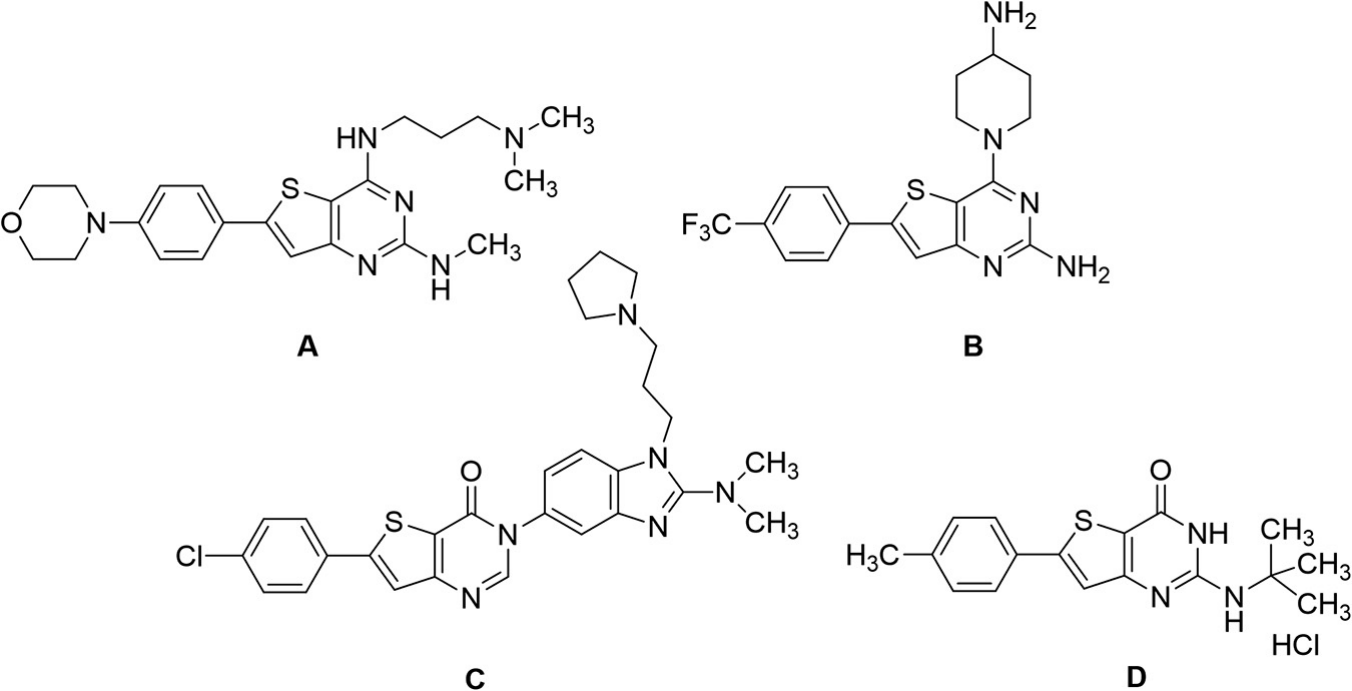
6-Phenylthienopyrimidine derivatives reported as antiplasmodial compounds. (A and B) 2,4-Diamino-6-phenylthieno[3,2-*d*]pyrimidine derivatives (27, 28). (C) 6-Phenylthieno[3,2-*d*]pyrimidinone derivative (26). (D) 2-Amino-6-phenylthieno[3,2-*d*]pyrimidinone derivative, gamhepathiopine (M1) (25).

Malaria remains the most prevalent tropical disease worldwide with a high mortality and morbidity: 229 million clinical cases and 409,000 deaths in 2019 according to the World Health Organization (WHO) (1). Beyond this human burden, malaria also has a dramatic social and economic impact on the development of 87 countries where malaria is endemic (1). Resistance to antimalarials, especially in Plasmodium falciparum, is the first serious problem that threatens to undermine the efficacy of all commercial antimalarial drugs, including the artemisinin components of the first-line artemisinin-based combination therapies (ACTs) that are recommended by the WHO (1–3). A second main issue is that very few antimalarial drugs are effective against the liver stage of the parasite, mainly 8-aminoquinolines (primaquine and tafenoquine) and atovaquone (naphthoquinone derivative). However, atovaquone tends to select resistant clones among blood stages of P. falciparum, while primaquine and tafenoquine carry a high risk of acute, life-threatening hemolysis in glucose-6-phosphate dehydrogenase (G6PD)-deficient patients, limiting the prophylactic use of these drugs (4–6). Moreover, Potter et al. demonstrated that primaquine is ineffective in people with low-metabolizing cytochrome P450 2D6 genotypes (7). A third main limitation to the chemotherapeutic fight against malaria is the fact that, among commercial antimalarial drugs that are available today, 8-aminoquinolines are the only ones able to block transmission by mosquito vectors, thanks to their activity on gametocytes, but are not clinically used for this indication (6). Thus, in May 2015, the WHO developed the Global Technical Strategy for Malaria 2016–2030 (8). Pillar 2 of this strategy is to accelerate efforts toward the elimination of malaria and attainment of malaria-free status. All evidence shows the fact that malaria elimination will require new strategies not only to target the hepatic stage, the erythrocytic stage, and the gametocyte development stages to prevent transmission to vectors but also to target the sporogonic stages inside the vector to prevent transmission to a new human host (2, 9, 10). The hepatic and sporogonic stages of the malaria parasite have remained largely underexploited as antimalarial targets, due to the poorly understood biology of these life cycle stages and the inherent technical difficulties in studying them (10, 11). Thus, the scientific community is nowadays actively developing and running drug-screening campaigns that target liver and/or sporogonic stages in addition to the erythrocytic stage (12, 13). Considering the drug candidates developed by Medicines for Malaria Venture (MMV) (https://www.mmv.org/research-development/mmv-supported-projects), these research efforts allowed four novel chemical entities (Fig. S1 in the supplemental material) displaying new mechanisms of action and multistage activities to reach clinical studies. Ganaplacide (KAF156) is an imidazolopiperazine derivative (14) that has reached phase II studies in combination with lumefantrine (15). Cipargamin (NITD609) is a spiro-indolone derivative that inhibits the P-type ATPase ATP4 of P. falciparum (*Pf*ATP4) (16) and is in phase II studies (17). MMV048 (MMV390048) is a bipyridine derivative that inhibits the P. falciparum phosphatidylinositol-4-kinase (*Pf*PI4K) (18) and is in phase II clinical studies (19). Finally, M5717 (DDD107498) is a quinoline derivative showing multistage activity (20) through the inhibition of the translocation factor eukaryotic elongation factor 2 (eEF2) and has reached phase I studies. Regarding the promising molecules that are studied at a preclinical stage, it appears that plasmodial kinases are quite promising targets (21, 22), as recently demonstrated by van der Watt et al. in the search for novel gametocytocidal compounds (23) or by Alam et al., who identified the P. falciparum cyclin-dependent-like protein kinase 3 (*Pf*CLK3) as a main drug target for developing novel multistage antimalarial drugs (24). In 2015, our group reported that a new compound from a chemical library containing kinase inhibitor candidates and belonging to the thienopyrimidinone series was active *in vitro* toward the blood stage of P. falciparum (50% effective concentration [EC_50_] =35 to 200 nM) and the liver stage of Plasmodium yoelii (EC_50_ = 35 nM) parasites infecting HepG2-CD81 cells (25). It was also shown that the mechanism of action (still under evaluation) of this new compound was not in common with the main ones described for commercial antimalarials (inhibition of heme crystallization, inhibition of P. falciparum dihydrofolate reductase [*Pf*DHFR], action on the mitochondrial membrane, and free radical production). This compound was named “gamhepathiopine” (denoted as “M1” herein) because of its multistage antiplasmodial activity that will be described in the present work: “gam” for its gametocytocidal activity, “hepa” for its activity on the hepatic stage, and “thiopine” as a contraction of thienopyrimidine. It is of note that several antimalarial thienopyrimidine derivatives, especially those bearing a phenyl substituent at position 6 of the scaffold, were reported in the literature (25–28), one of them being presented as a potential *Plasmodium* kinase inhibitor (26); their structures are presented in Fig. 1. We decided to extend the study of the antimalarial activity of M1 toward (i) hepatic stages of P. yoelii, Plasmodium cynomolgi, and P. falciparum in murine, simian, and human primary hepatocytes *in vitro*, along with P. yoelii *in vivo*, (ii) gametocyte induction development and exflagellation, (iii) *in vivo* transmission blocking, and (iv) artemisinin-resistant P. falciparum parasites in the proliferation or quiescent state, to evaluate the global potential of this new chemotype.

## RESULTS

### *In vitro* experiments. (i) Inhibition of the liver forms of *Plasmodium*.

*In vitro* liver-stage assays were conducted to determine if M1 possessed activity against *Plasmodium* parasites. M1 was found to be quite active against the liver stage of P. yoelii, P. falciparum, and P. cynomolgi *in vitro*. As shown by the data in Table 1, the activities of M1 (EC_50_ = 0.45 to 1.5 μM) were very close to those of primaquine (EC_50_ = 0.6 to 2.7 μM). Regarding P. falciparum in human hepatocytes and P. cynomolgi in simian hepatocytes, the liver-stage development was fully inhibited from 14 μM (EC_50_ = 450 nM and 1,500 nM, respectively). Nevertheless, at all tested concentrations (0.1 to 50 μM), M1 was not active on P. cynomolgi hypnozoites. This inhibition property is unlikely to result from a cytotoxic effect of M1 on hepatic cells, as the drug concentration affording 50% cell viability (50% cytotoxic concentration [CC_50_]) (Table 1) was found to be higher than 60 μM toward all primary hepatocytes (murine, simian, and human), a concentration higher than most used in the experiments.

**TABLE 1:** Gamhepathiopine shows submicromolar activities on *Plasmodium* hepatic stages

*Plasmodium* species (type of hepatocytes used)^*a*^	Value against liver-stage parasites^*b*^					
	Gamhepathiopine (M1)			Primaquine (control)		
	EC_50_ (μM)	SI	Size reduction at 10 μM (%)	EC_50_ (μM)	SI	Size reduction at 10 μM (%)
P. yoelii (MH)	0.45 ± 0.1	>133	100	0.64 ± 0.1	110	ND
P. falciparum (HH)	0.45 ± 0.15	>133	80	0.6 ± 0.06	66	100
P. cynomolgi (SH)	1.5 ± 0.3	>40	80	2.72 ± 0.34	12	70

a MH, murine primary hepatocytes; HH, human primary hepatocytes; SM, simian primary hepatocytes.

b EC_50_s are expressed as mean values ± standard errors of the means of triplicate experiments (each concentration in triplicate). SI, selectivity index, calculated as hepatocyte CC_50_/EC_50_. CC_50_ values of M1 were >60 μM for all primary hepatocytes.

**FIG 2:**
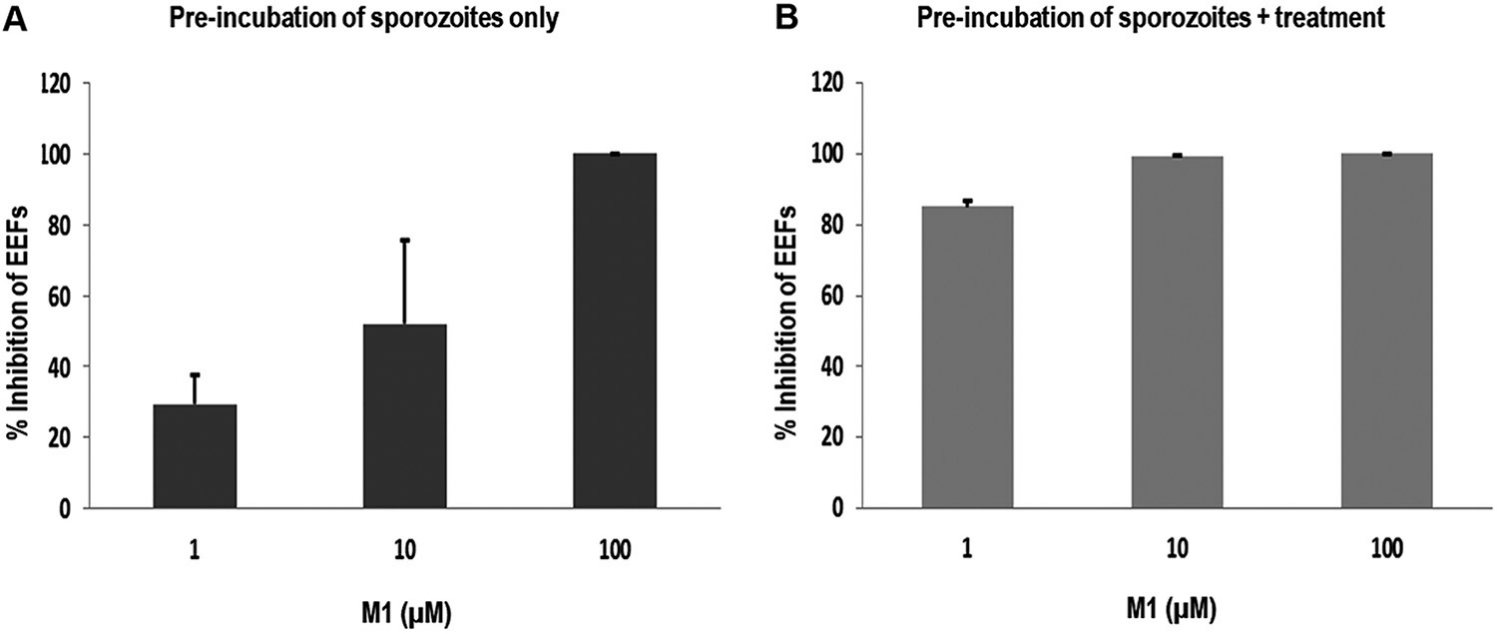
Gamhepathiopine inhibits hepatocyte infection. Effect on hepatocyte invasion by P. falciparum sporozoites. Sporozoites were preincubated with the drug at 1, 10, or 100 μM for 1 h at room temperature prior to being added to hepatocytes. Three hours later, after penetration of sporozoites into hepatocytes, cultures were washed and incubated in the presence of medium without M1 (A) and with M1 (B) at 1, 10, or 100 μM every day until day 6. EEFs, exoerythrocytic forms. The results are expressed as mean values ± standard errors of the means of duplicate experiments (testing of each concentration was performed in triplicate). The reference compound used was primaquine, for which the percentage of inhibition was 100% at all concentrations tested.

In another approach, we evaluated the effect of M1 on parasite size (μm^2^). Comparison of the sizes of schizonts developing in primary hepatocyte cultures after infection showed that M1 significantly reduced the size of the parasites (Table 1). The effects of primaquine and M1 on parasite size were significant only from 10 μM. At this concentration, M1 led to size reductions of 100% for P. yoelii and 80% for both P. falciparum and P. cynomolgi, while primaquine led to a 100% reduction for P. falciparum and 70% for P. cynomolgi. In order to determine the effect of M1 on the invasion of hepatocytes by sporozoites, we exposed P. falciparum sporozoites to 1, 10, and 100 μM drug for 1 h at room temperature before incubating them with primary human hepatocytes. Three hours later, after sporozoite penetration into hepatocytes, cultures were washed and further incubated with or without the presence of the drug for 6 days (Fig. 2). Our results indicated that M1 inhibited the invasion of sporozoites into hepatocytes in a dose-dependent manner: 30% at 1 μM, 55% at 10 μM, and close to 100% at 100 μM. When cultures were additionally treated, the inhibition of invasion was quite good, varying from 80% at 1 μM to 100% at both 10 μM and 100 μM.

### (ii) Transmission blocking effect: gametocytocidal activity assays.

**FIG 3:**
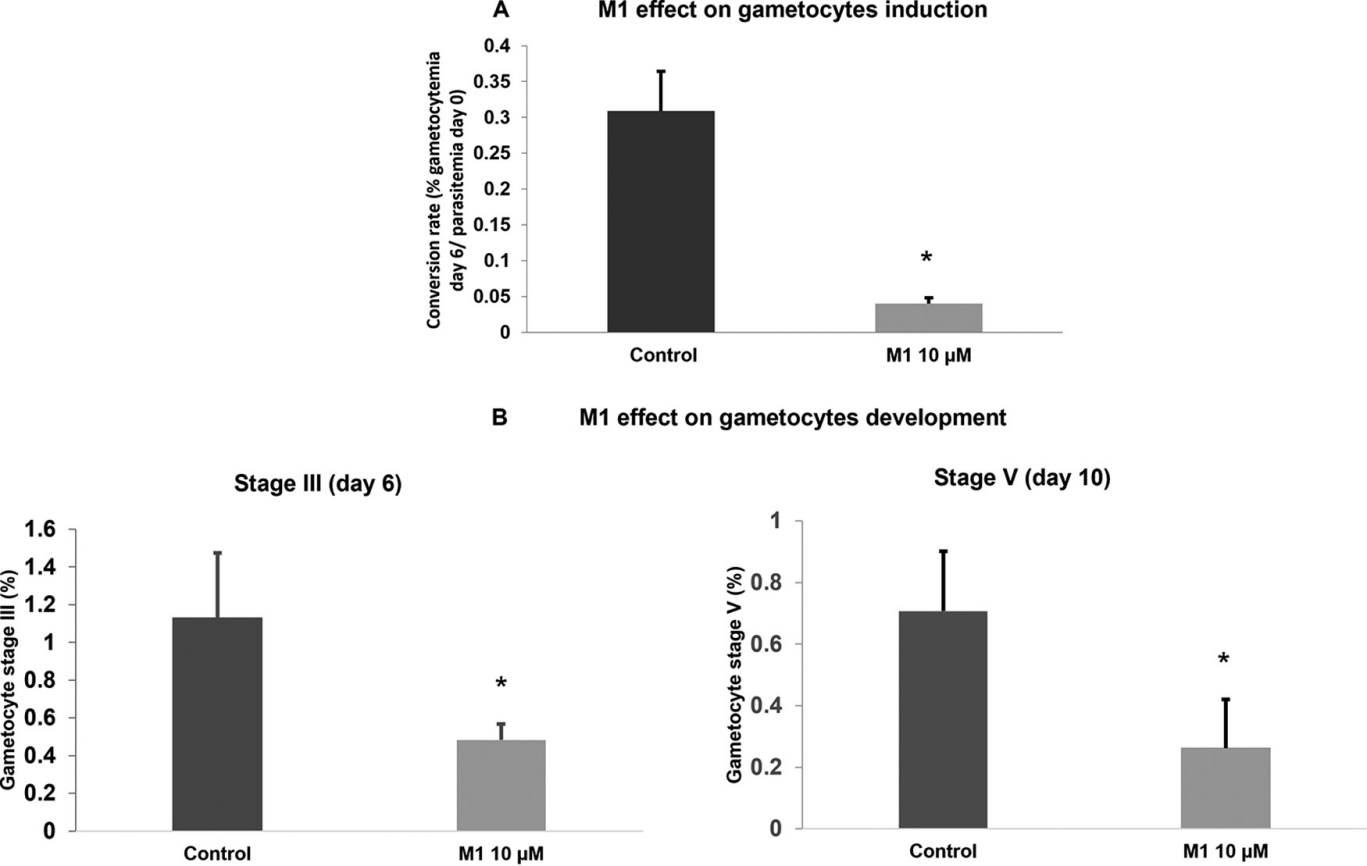
Gamhepathiopine inhibits both induction and development of gametocytes. Effects of gamhepathiopine (M1) *in vitro* on gametocytogenesis. (A) Effect of gamhepathiopine *in vitro* on P. falciparum gametocyte induction. The drug and NAG were added to the culture simultaneously at day zero. The control had DMSO added instead (untreated). We estimated the conversion rate (% gametocytemia at day 6/% parasitemia at day zero) in gametocytes (*; *P* = 0.0209). (B) Effect of gamhepathiopine *in vitro* on P. falciparum gametocyte development. Stage III gametocytes at day 6 (*, *P* = 0.0465), and stage V gametocytes at day 10 (*, *P* = 0.0122). The control had DMSO added instead (untreated). The results for gametocytemia and parasitemia are expressed as mean values ± standard errors of the means of duplicate experiments.

Sexual-stage gametocytes are responsible for the transmission of malaria parasites from the infected host to the mosquito vector. Only 8-aminoquinolines are proven effective at eliminating mature gametocytes (5). To examine whether M1 possessed activity against the sexual stage of P. falciparum, we conducted several assays (Fig. S2). In the first assay, M1 (10 μM) was added at day zero (D0) along with *N*-acetylglucosamine (NAG) for gametocyte induction to evaluate the conversion rate of the asexual parasites into gametocytes. In the second assay, M1 (10 μM) was added at D3 after gametocyte induction, to observe the effect on gametocyte development. In these two assays, the treatment was renewed every day until D10. In the last assay, at D10, before induction of gamete formation, mature gametocytes were preincubated with M1 (10 μM) for 2 or 24 h. Our results showed that M1 had the ability to reduce the conversion rate of asexual parasites into gametocytes by 87.8% (Fig. 3A). M1 also influenced the development of gametocytes when used after the induction. At days 6 and 10 postinduction, corresponding to gametocyte stages III and V, respectively, we noticed a difference between the control and the culture treated with M1: the stage III mean values were 1.29 for the control and 0.48 for M1 (*P* = 0.0465), and the stage V mean values were 0.70 for the control and 0.26 for M1 (*P* = 0.0122) (Fig. 3B).

**FIG 4:**
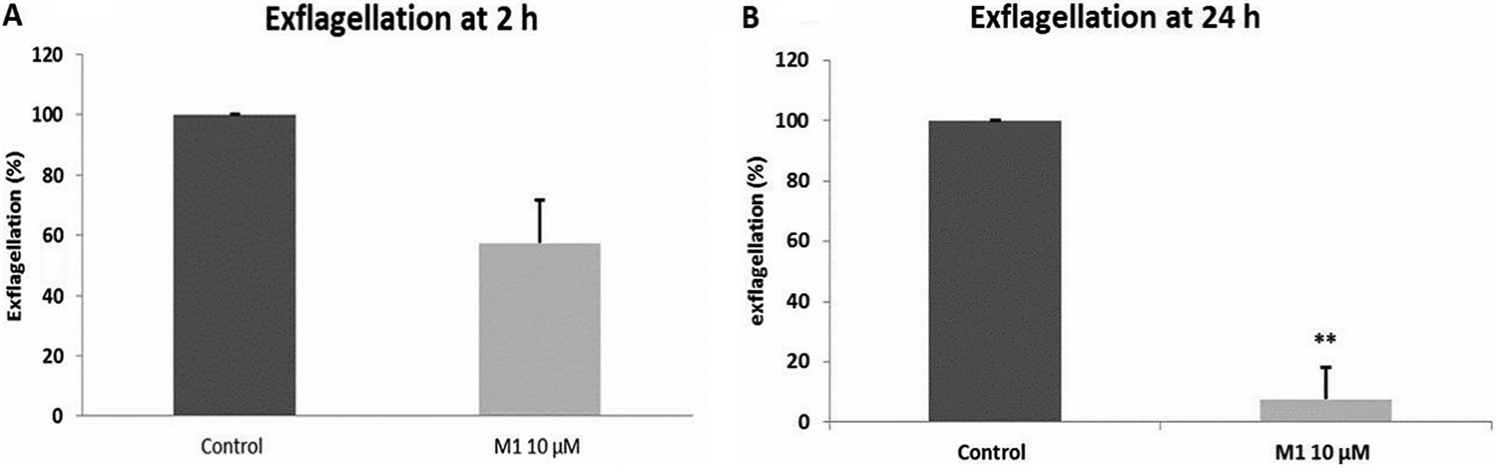
Gamhepathiopine inhibits the exflagellation of gametocytes. Effect of M1 *in vitro* on P. falciparum gametocytes. (A) Effect on the exflagellation of mature gametocytes (stage V) after 2 h of treatment with M1 (10 μM). The results are expressed as mean values ± standard errors of the means of 3 independent experiments. (B) Effect on the exflagellation of mature gametocytes (stage V) after 24 h of treatment with M1 (10 μM). The control was treated with DMSO. **, *P* = 0.065. The results are expressed as mean values ± standard errors of the means of duplicate experiments. For all experiments, each point was counted in triplicate.

We also investigated the effect on mature stage V gametocyte development into gametes. The exflagellation-blocking assays were performed in triplicate. The mean percentages for male exflagellation were 57% at 2 h and 7.5% at 24 h (Fig. 4). These results suggested that M1 inhibited exflagellation, either interfering directly during the process of gamete formation or “sterilizing” the mature gametocyte in such a way that it was metabolically viable but somehow unable to form gametes.

### (iii) Activity toward artemisinin-resistant P. falciparum strains.

**FIG 5:**
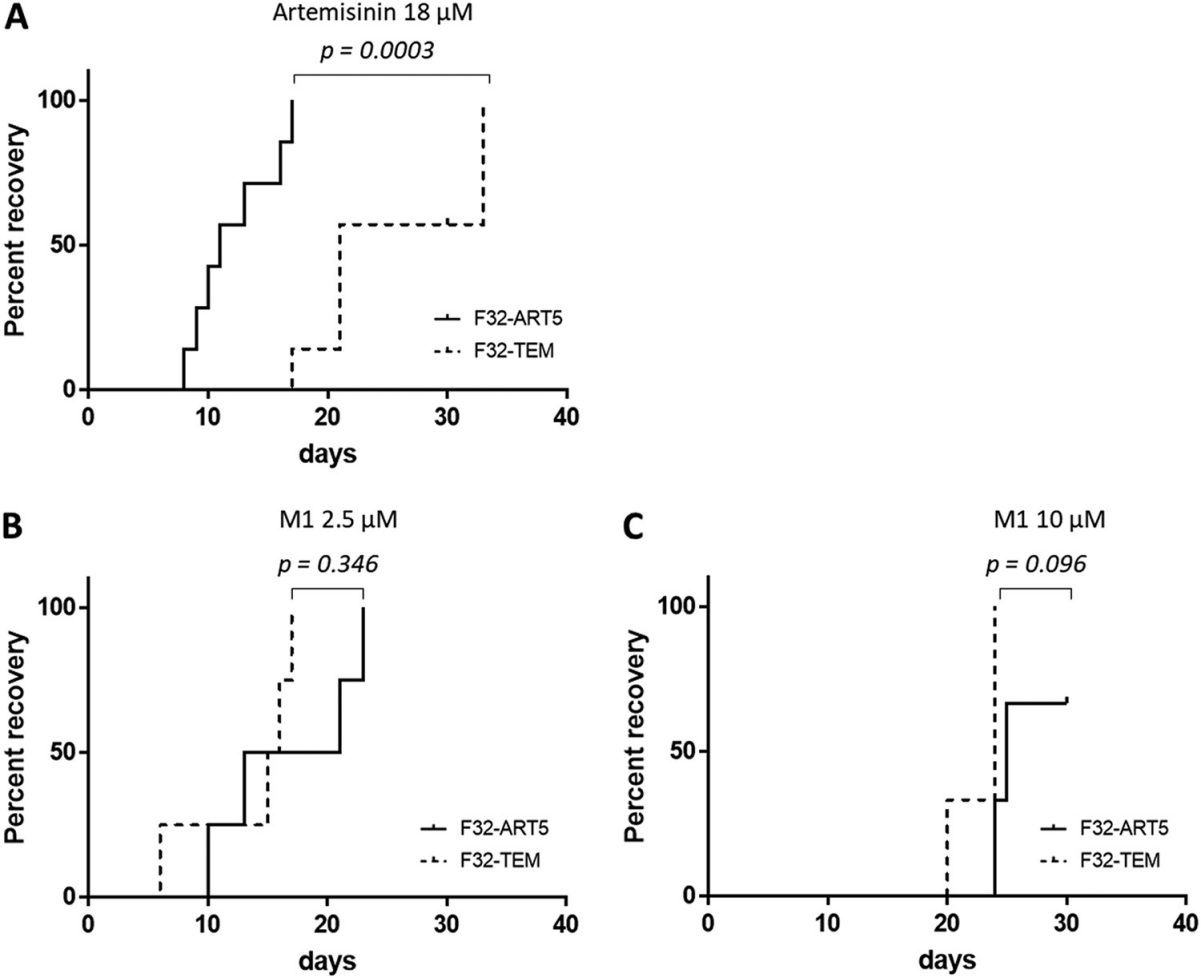
Absence of cross-resistance between M1 and artemisinin. Recrudescence curves of F32-ART5 and F32-TEM parasites after a 48-h drug exposure using Kaplan-Meier survival analysis. The final event was defined as the time necessary for P. falciparum cultures to reach initial parasitemia. Observations were considered censored if no recrudescence was observed at day 30. Log-rank test was performed (*P* < 0.05 is considered significant). (A) Treatment with 18 μM artemisinin (*n* = 7). (B) Treatment with 2.5 μM M1 (*n* = 4). (C) Treatment with 10 μM M1 (*n* = 3).

**FIG 6:**
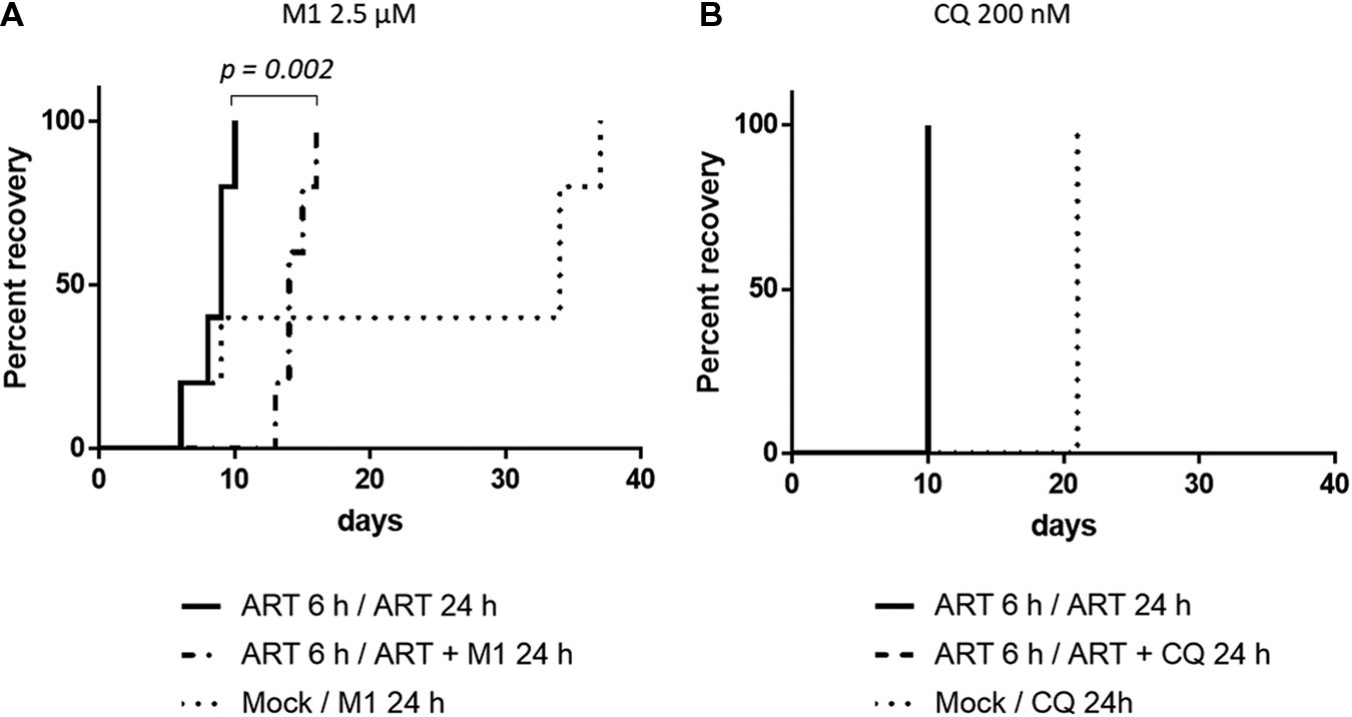
Activity of M1 against artemisinin (ART)-resistant parasites at quiescent stage. Recrudescence curves of F32-ART5 parasites were tested by QSA using Kaplan-Meier survival analysis. The final event was defined as the time necessary for parasite cultures to reach initial parasitemia. Observations were considered censored if no recrudescence was observed at day 30. Log-rank test was performed (*P* < 0.05 was considered statistically significant). (A) Treatment with 2.5 μM M1 (*n* = 5). (B) Treatment with 200 nM CQ (*n* = 1). Recrudescence curve of parasites treated with the combination artemisinin/artemisinin + CQ overlaps that of the parasites treated with artemisinin alone.

As shown by the data in Table 2, M1 showed high antiplasmodial activity against both P. falciparum F32-ART5 and F32-TEM lineages, with EC_50_s ranging from 227 to 283 nM. The M1 EC_50_s did not differ significantly between these two laboratory strains. However, despite the artemisinin-resistant genotype of F32-ART5 (29), the EC_50_s of artemisinin were similar for both strains, due to the specific P. falciparum quiescence-based mechanism of resistance to artemisinins, leading to parasite cell cycle arrest during drug exposure (30–32). These data confirmed that the standard proliferation chemosensitivity assay is not appropriate for the detection of either artemisinin resistance or cross-resistance with artemisinin, as already reported (30–32). In contrast, artemisinin resistance can be evidenced *via* the recrudescence assay, with a faster recrudescence for F32-ART5 than for F32-TEM parasites after 48 h of artemisinin exposure (*P* = 0.0003) (Fig. 5A). Interestingly, M1 presented no cross-resistance with artemisinin, since there was no significant difference in recrudescence times between the F32-TEM and F32-ART5 lines after 48 h of exposure to M1 (*P* = 0.346 at 2.5 μM, and *P* = 0.096 at 10 μM) (Fig. 5B and C). Moreover, M1 showed activity against quiescent parasites. Indeed, when quiescence was induced by a 6-h pretreatment with 3 μM artemisinin before the addition of the drug to be tested, the recrudescence time of F32-ART5 quiescent parasites treated with M1 was significantly different (*P* = 0.002) from the recrudescence time of F32-ART5 quiescent parasites treated with artemisinin alone, with a median delay in recrudescence time of 6 days (Fig. 6A). In contrast, when the tested molecule was inactive against parasites, like chloroquine (CQ) (33), there was no difference in the recrudescence times of parasites treated with the combination of artemisinin/artemisinin plus CQ and of parasites treated with artemisinin alone, although the drug was active against proliferating parasites (Fig. 6B).

**TABLE 2:** Gamhepathiopine is active against artemisinin-resistant parasites^*a*^

Treatment	Mean EC_50_ ± SD (nM)^*b*^		*P* value^*c*^
	F32-ART5	F32-TEM	
M1	227 ± 40	283 ± 125	0.547
Artemisinin	11 ± 0.5	11.1 ± 1.6	0.894

a Chemosensitivity to M1 was determined by standard assay of asexual blood-stage P. falciparum F32-ART5 and F32-TEM lineage parasites. Artemisinin was used as the control drug.

b Results represent the values for three independent experiments.

c Paired *t* test. A *P* value of <0.05 was considered statistically significant.

### *In vivo* experiments. (i) Inhibition of the liver forms of P. yoelii in a mouse model.

**FIG 7:**
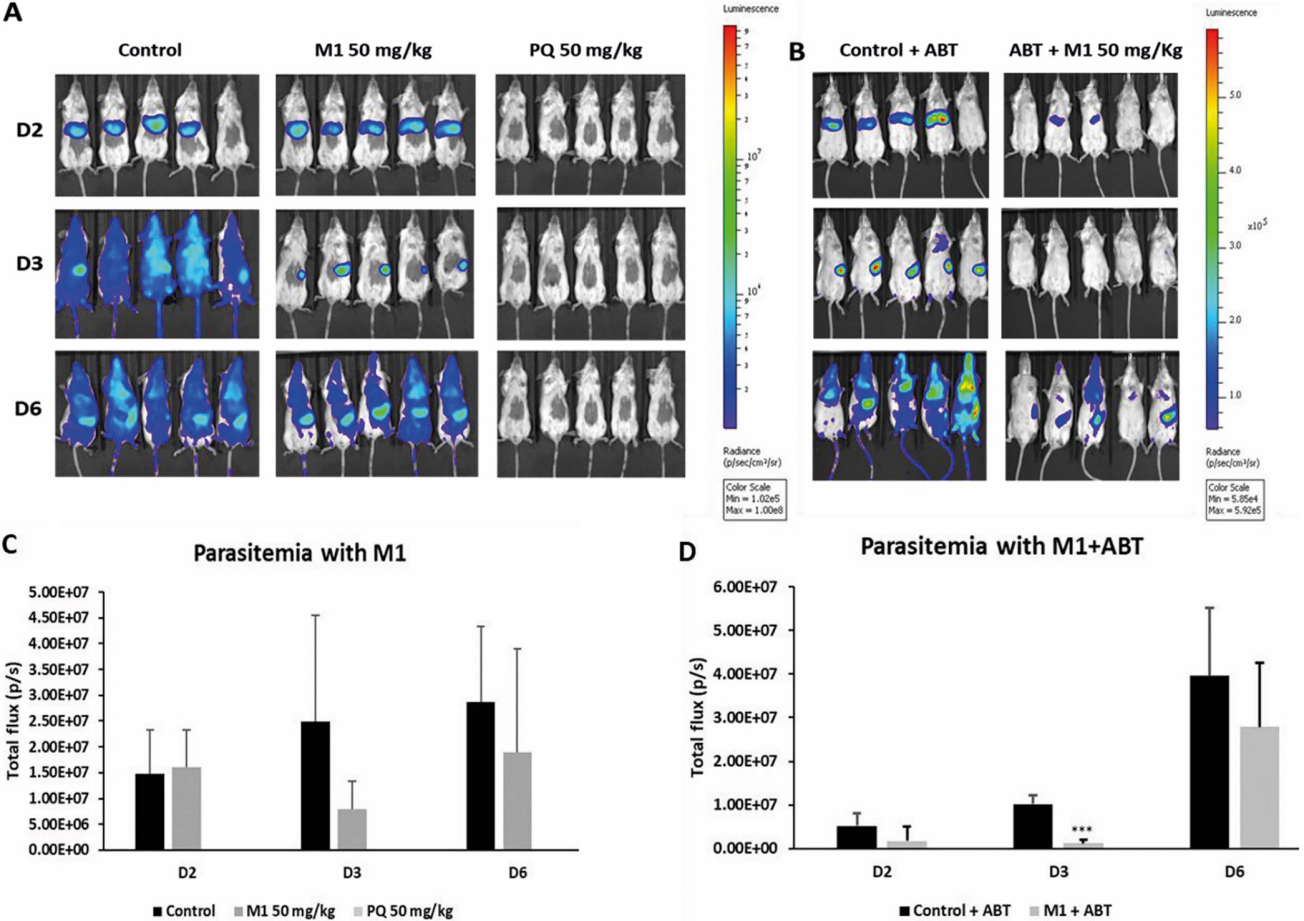
*In vivo* antimalarial activity of M1 against liver and blood stage is limited by its microsomal hepatic metabolism. Mice were infected with 10,000 sporozoites. M1 (50 mg/kg) and primaquine (PQ) (50 mg/kg) were administered on days −1, 0, and 1 to monitor activity against liver stage and on days 3 and 4 to monitor blood-stage activity. (A) Representative *in vivo* images (IVIS Spectrum) of luminescence in the liver of live BALB/c mice at different time points. Rainbow images show the relative levels of luminescence, ranging from low (blue), to medium (green), to high (yellow/red). (B) Activity of M1 with 1-aminobenzotriazole (ABT). (C) Activity of M1 and PQ. Luminescence levels (photons/s) of each group at D2, D3 and D6 (mean ± SD). (D) Activity of M1 and ABT. Luminescence levels (photons/s) of each group at D2, D3, and D6 (mean ± SD). ***, *P* < 0.0056. The control group was treated with DMSO.

In an *in vivo* liver-stage assay, M1 was administered to mice at 50 mg/kg of body weight intraperitoneally (i.p.), and liver-stage development was assessed at 44 h postinfection (p.i.) on day 2. The results showed that, contrary to the data obtained *in vitro*, M1 did not prevent the development of liver-stage parasites *in vivo* at day 2 (Fig. 7A). Thus, at D3 and D6, blood-stage parasites were observed in the bloodstream. However, the time of appearance of the parasites was delayed in the group treated with M1 (to D4 instead of D3 for the control). In M1-treated mice, parasites were retained at the level of the spleen at days 3 and 4 (Fig. 7A). All mice were followed for the development of the blood-stage infection. Parasitemia on days 3 and 6 post-sporozoite infection was determined on blood smears. The results indicated that M1 reduced the burden of blood-stage parasites (Fig. 7C). Mice treated with control compound primaquine (50 mg/kg) remained clear of parasitemia throughout the assay. To understand the discrepancy between the *in vitro* and *in vivo* efficacies of M1 against the hepatic stage, the same experiment was done on mice in which P450 cytochromes were inhibited by the i.p. administration of 1-aminobenzotriazole (ABT) at 50 mg/kg (34, 35) from D1 to D3. The results presented in Fig. 7B and D showed that, with ABT cotreatment, a significant statistical difference was observed (*P < *0.0065) between the luminescence levels of the control group and the treated group: the *in vivo* activity of M1 against the hepatic stages was improved when inhibiting P450 cytochromes with ABT cotreatment but did not allow full eradication of hepatic schizonts under the conditions tested.

### (ii) Transmission-blocking evaluation: mosquito feeding assay.

**FIG 8:**
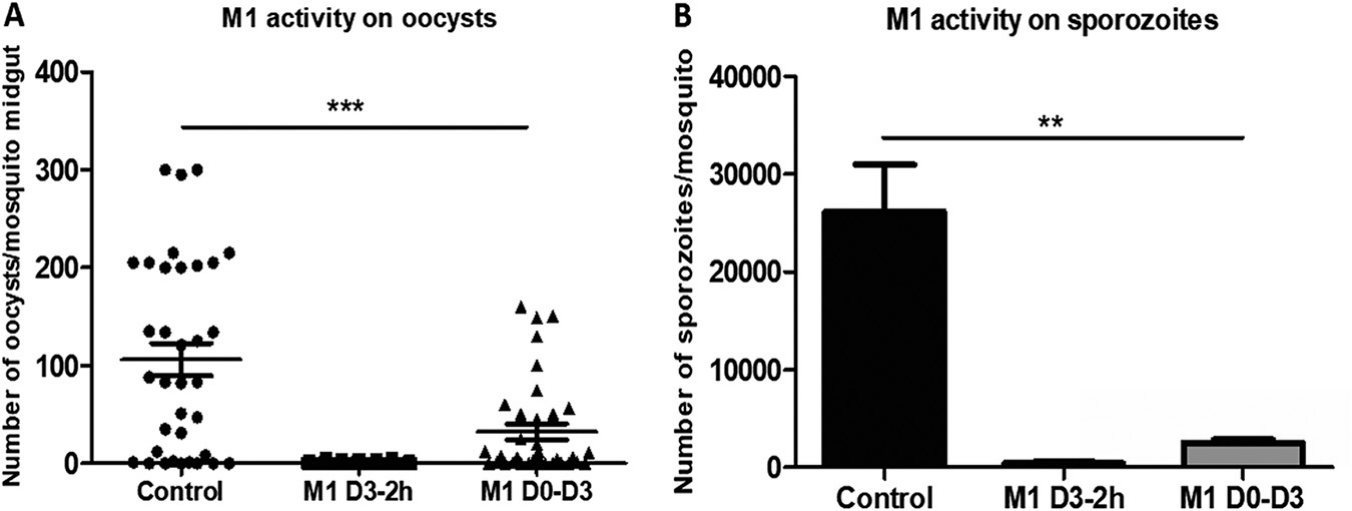
Treatment of infected mice with 50 mg/kg gamhepathiopine inhibits sporogonic cycle in mosquitos. Mice from the group whose results are labeled M1 D3-2h received the treatment on D3, 2 h before mosquito feeding. Mice from the group whose results are labeled M1 D0-D3 received the treatment every day from D0 to D3. (A) Number of oocysts per midgut. Each point represents the number of oocysts from an individual mosquito. Oocyst density in M1 is significantly different from that in the untreated group (***, *P* < 0.0001). (B) Number of sporozoites extracted from salivary glands of mosquitoes (total number of sporozoites/number of mosquitoes) (* *P* < 0.0265). The results shown are those of one representative assay among the three independent assays performed. Results are expressed as the mean of three sporozoite counts ± SD.

M1 was tested for its ability to inhibit oocyst formation in a rodent malaria model. On day 4 after the injection of 10^7^ infected red blood cells, mice treated with M1 at 50 mg/kg during D0 to D3 showed a 50% reduction in parasitemia before mosquito feeding (Table 3). The mouse group treated with M1 at 50 mg/kg at day 3, 2 h before mosquito feeding, had levels of parasitemia and gametocytemia similar to those in the untreated group (Table 3). Two hours after this treatment, the mosquitoes were allowed to feed on mice. The results showed that M1 reduced the infection rate (the percentage of mosquitoes with oocysts) from 82.8% (untreated) to 37.1%. Among the infected mosquitoes, M1 affected the sporogonic development of P. yoelii. The mean oocyst density (oocyst burden) was 106 oocysts per mosquito, with a range of 0 to 300 oocysts per mosquito. M1 demonstrated potent activity, up to a 95% reduction of the total oocyst count relative to the count in the control group (*P < *0.0001), as shown by the data in Table 3 and Fig. 8A. The effect of M1 appeared stronger when treating mice just before (2 h) mosquito feeding. The reduction of the mean oocyst density logically led to a major decrease in the numbers of sporozoites in the M1 groups compared to the numbers in the untreated group (Fig. 8B).

**TABLE 3:** Treatment of infected mice with gamhepathiopine inhibits gametocytogenesis and reduces mosquito infection^*a*^

Parameter	Value for indicated treatment group		
	Untreated	M1 D3 2 h	M1 D0–D3
Day 4 p.i. in mice prior to mosquito feeding			
Mean % parasitemia ± SD	0.97 ± 0.17	0.99 ± 0.34	0.47 ± 0.24
Mean % gametocytemia ± SD	0.05 ± 0.004	0.04 ± 0.01	0.04 ± 0.009

Sporogonic stage in fed mosquitoes			
No. of mosquitoes infected/total no. of mosquitoes (%)	29/35 (82.8)	13/35 (37.1)	25/35 (71.4)
No. of oocysts per mosquito	106	1	32

a M1 was administered at 50 mg/kg i.p. Mice in the M1 D3 2 h group received the treatment on day 3, 2 h before mosquito feeding. Mice in the M1 D0–D3 group received the treatment every day from D0 to D3 before mosquito feeding. Parasitemia and gametocytemia before mosquito feeding and the prevalence of infected mosquitoes in each group were determined.

### Microsomal stability, metabolism, and metabolites of gamhepathiopine.

When incubated with female mouse microsomes, gamhepathiopine (M1) showed a poor stability, with a half-life (*t*_1/2_) of 11 min. Searching for the microsomal metabolites of M1 by incubating M1 with female mouse microsomes and analyzing the resulting medium by liquid chromatography-tandem mass spectrometry (LC-MS/MS), it appeared that 2 sites of M1 were targeted by the P450 cytochromes: the tolyl (methylphenyl) moiety and the *tertio-*butyl group were oxidized, successively leading to alcohol, aldehyde, and carboxylic acid derivatives. The metabolites formed on the tolyl moiety that were proposed by the LC-MS/MS analysis were synthesized, using a new optimized organic synthesis pathway (Fig. S4). The preparation of these 3 metabolites (numbered **7**, **8**, and **10** herein) allowed the confirmation of the structures proposed by the LC-MS/MS analysis (confirmation of retention times and fragmentations) and evaluation of their *in vitro* activity on the blood stage of P. falciparum (strain K1), along with the hepatic stage of P. yoelii in HepG2-CD81 cells for metabolites **7** and **10**. As shown by the data in Table 4, all metabolites (especially carboxylic acid **10**) were less active (1- to 2-log increases in the EC_50_) than M1 against both the blood and hepatic stages.

**TABLE 4:** Gamhepathiopine microsomal metabolites lose antiplasmodial activity^*a*^

Molecule or drug^*b*^	Structure	EC_50_ (μM) for:		CC_50_ (μM) for HepG2 cells
		P. falciparum strain K1 in RBC	P. yoelii in HepG2-CD81 cells	
Gamhepathiopine (M1)	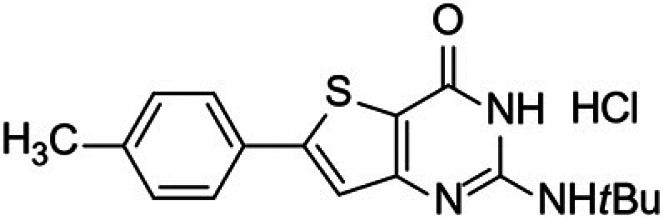	0.045	0.067	24.0
Metabolite **7**	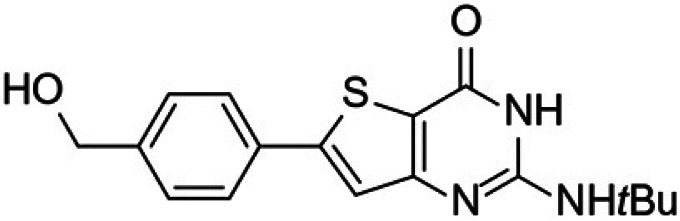	0.4	1.0	25.0
Metabolite **8**	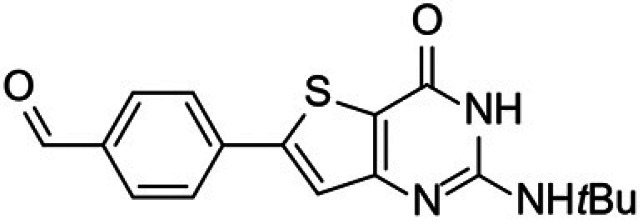	3.1	ND^*c*^	25.3
Metabolite **10**	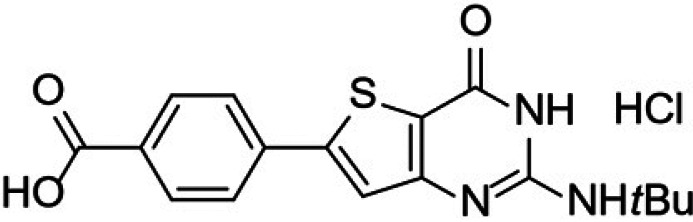	>10^*d*^	>10^*d*^	>10^*d*^
Atovaquone		0.001	0.003	>15.6^*d*^
Chloroquine		0.8		30
Doxycycline		6		20
Primaquine			3.6	
Doxorubicin				0.2

a *In vitro* activities of gamhepathiopine (M1) and some of its metabolites (**7**, **8**, and **10**) against the erythrocytic stage of P. falciparum (K1) and hepatic schizonts of P. yoelii in HepG2-CD81 cells and corresponding cytotoxicities on the human HepG2 cell line.

b Atovaquone, chloroquine, doxycycline, and primaquine were used as positive antimalarial controls. Doxorubicin was used a positive cytotoxic control.

c ND, not done.

d The EC_50_ or CC_50_ value could not be reached because of a lack of solubility in the culture medium.

### Complementary *in vitro* toxicity evaluations.

Finally, to complete the *in vitro* safety study of M1, its cytotoxicity on the canine renal MDCK (Madin-Darby canine kidney) cell line was also evaluated, indicating a low cytotoxicity with a CC_50_ value of 39.5 μM. In parallel, to evaluate possible cardiac toxicity, the binding of M1 toward the hERG channel was determined, showing no affinity (Fig. S3) and, consequently, a safe profile.

## DISCUSSION

To realize the goal of malaria eradication, identification of new potent compounds active against multiple life stages of the parasite is necessary. Because most of the currently licensed antimalarials only target the asexual intraerythrocytic stage, we urgently need to expand our antimalarial arsenal. Beyond efficacy against blood stages, drugs that can effectively target the hepatic stage and gametocyte stages, responsible for the transmission to the mosquito vector, will be required for malaria elimination (2). Furthermore, in the context of artemisinin resistance, these new drugs must be active against artemisinin-resistant parasites, including quiescent ones.

In this demanding context, the data presented herein demonstrated that gamhepathiopine (M1), a potent (P. falciparum strain K1 EC_50_ = 45 nM) antiplasmodial molecule toward the erythrocytic stage (25), also displayed *in vitro* activity against artemisinin-resistant quiescent parasites, was active against the liver stage of P. falciparum in human primary hepatocytes, inhibited the formation and development of gametocytes, and showed transmission-blocking activity.

### *In vitro*, gamhepathiopine showed activity toward the liver stages of *Plasmodium* spp.

We first conducted *in vitro* assays against the liver stages of P. yoelii, P. falciparum, and P. cynomolgi in corresponding primary hepatocytes. As shown by the data in Table 1, the activities of M1 were similar to those of primaquine (EC_50_s of 450 to 1,500 nM for M1 versus 600 to 2,720 nM for primaquine). M1 acted both on the number of parasites and also on their development, by reducing their size. As the number of parasites developing in hepatocytes depends on the number of sporozoites that can invade them, we studied the effect of M1 on the penetration of sporozoites into hepatocytes and their development there. Incubation of sporozoites with M1 reduced the number of parasites by 30% at 1 μM and 60% at 10 μM (Fig. 2A), whereas complete inhibition of sporozoite invasion was achieved by treating both sporozoites and infected hepatocyte cultures with M1 at 10 μM (Fig. 2B). This action against sporozoites, coupled with that against the hepatic stages, makes M1 an interesting model for developing novel antimalarials with chemoprophylaxis potential.

### *In vitro*, gamhepathiopine inhibited the induction and development of gametocytes along with the exflagellation of gametes.

Antimalarials that prevent the transmission of malaria parasites (from human host to mosquito vector or from mosquito to human) have an important role to play in malaria control programs and may help to contain the spread of drug resistance (36). In fact, at present, a single low dose of 0.25 mg/kg of primaquine remains the only gametocytocidal treatment recommended by the WHO. Unfortunately, its deployment is limited because of concerns over hemolytic effects in patients with glucose-6-phosphate dehydrogenase (G6PD) deficiency. To explore the potential transmission-blocking activity of M1, we investigated its ability to (i) prevent the induction of gametocytes, (ii) affect their development, and (iii) disturb exflagellation, following a protocol using P. falciparum gametocyte cultures that is summarized in Fig. S2. As shown by the data in Fig. 3, M1 used at 10 μM was a significant inhibitor of gametocyte induction. Then, early-stage gametocytes (stages I and II) were exposed to M1 at 10 μM. As shown by the data in Fig. 3A and B, M1 significantly inhibited the development of gametocytes at both day 6 and 10. Moreover, when exposing stage V gametocytes to M1 for 2 or 24 h, inhibition of the rate of exflagellation was observed (Fig. 4). These data highlighted M1 as a transmission-blocking drug with inhibitory activity on more than one critical step in the sexual stages of the parasite life cycle.

### *In vitro*, gamhepathiopine was active on artemisinin-resistant and quiescent parasites.

In the context of a worldwide threat of artemisinin resistance spreading, it is essential to assess the efficacy of new antimalarial compounds toward this very special drug resistance. M1 exerted similar activities against P. falciparum artemisinin-resistant and artemisinin-sensitive parasites (F32-ART5 and F32-TEM, respectively) as evaluated by a standard chemosensitivity assay (Table 2). Very interestingly, M1 presented no cross-resistance with artemisinin, whatever the dose tested (Fig. 5), and showed activity against artemisinin-resistant parasites in the quiescent state (Fig. 6), contrary to the case for several antimalarial drugs (31, 33). A drug bearing such properties could be a gold standard partner in ACTs, allowing counteraction of artemisinin resistance and avoiding the risk of selection of partner drug resistance.

### *In vivo*, gamhepathiopine showed transmission-blocking activity but was rapidly metabolized by P450 cytochromes into inactive metabolites.

It was previously reported (25) that M1, administered twice a day at 2.5 mg/kg for 3 days to mice infected by Plasmodium berghei, was moderately active in comparison with chloroquine used at the same dose and an untreated group (parasitemia at day 4 was 9% in the untreated group, 4.9% in the M1 group, and 1.5% in the chloroquine group). This *in vivo* lack of efficacy was confirmed when studying the activity of M1 against the liver stage of P. yoelii. Effectively, when infecting mice with sporozoites on day zero, we noted a non-statistically significant decrease at day 3 in the number of hepatic parasites in the group treated with M1 (versus untreated group), but M1 was not efficient enough to clear hepatic parasites, meaning that erythrocytic schizogony could take place, leading to a high level of parasitemia at day 6, contrary to what was observed in the primaquine group (Fig. 7A and C). By repeating the same experiment after treating mice with 1-aminobenzotriazole (ABT), a multi-P450 CYP inhibitor (34, 35), the efficacy of M1 was improved at days 2, 3, and 6 (Fig. 7B and D), appearing statistically significant at D3, confirming that the loss of *in vivo* efficacy of M1 was probably due to its metabolization by hepatic cytochromes into inactive derivatives.

Effectively, in an *in vitro* microsomal stability assay, M1 presented a short half-life of 11 min. To validate this hypothesis, the metabolites of M1 formed after incubation with mouse microsomes were first searched by LC-MS/MS, revealing 2 metabolic hot spots in the structure of M1: the tolyl (methylphenyl) and *tertio*-butyl moieties that could afford oxidized metabolites (alcohols, aldehydes, and carboxylic acids). Then, 3 of the suspected metabolites were synthesized (Fig. S4), allowing confirmation of the proposed structures, and were evaluated for their *in vitro* antiplasmodial activity toward the erythrocytic K1 P. falciparum strain and hepatic stage of P. yoelii in HepG2-CD81 cells (Table 4). In comparison with M1, alcohol **7**, aldehyde **8**, and carboxylic acid **10**, microsomal metabolites of M1 on the tolyl moiety, showed reduced to abolished antiplasmodial activities (EC_50_s of 0.4 to >10 μM), proving that the partial *in vivo* activity of M1 was due to its rapid metabolization into less-active and inactive derivatives and arguing for the conception of new M1 derivatives bearing metabolic blocker groups on the 2 metabolized sites of the pharmacophore.

Finally, we evaluated the effect of M1 on the formation of P. yoelii oocysts in Anopheles stephensi mosquitoes (37) *via* a feeding assay on infected mice. The presence of M1 in the infectious blood feed had a significant impact on the mean number of oocysts per mosquito (Table 3 and Fig. 8). In view of the results obtained *in vivo* showing the metabolization of M1, the tests were carried out by treating the mice either from D0 to D3 or 2 h before feeding the mosquitoes in order to cancel the effect of the metabolization. The prevalence of oocyst infections in the control group was 82.8%, yielding a geometric mean of 106 oocysts/mosquito (Table 3). With 95% reduction of the total oocyst count relative to the count in controls, M1 reduced the oocyst burden in mosquito midguts. The reduction in the number of oocysts inevitably led to the reduction of sporozoites (Fig. 8). This reduction was more marked when the treatment with M1 was done only 2 h before feeding the mosquitoes than in the group treated from D0 to D3, further highlighting the metabolism of M1 into inactive derivatives.

### *In vitro* toxicity profile of M1.

Assessment of the toxicity of M1 toward the MDCK (Madin-Darby canine kidney) cell line (CC_50_ = 39.5 μM) and primary hepatocytes (CC_50_ > 60 μM in murine, simian, and human hepatocytes) showed a safe profile. Moreover, *in vitro*, M1 did not bind to the hERG channel, indicating a good profile toward cardiotoxicity. These new data were in accordance with previously reported *in vitro* evaluations regarding HepG2 or CHO cell lines (CC_50_ ≥ 25 μM), negative Ames test, or negative DNA-methyl green assay (25).

In conclusion, these results indicate that the thienopyrimidinone scaffold is a novel antiplasmodial chemotype showing great potential for the design of new antimalarial drugs with multistage activity. Effectively, in addition to previously reported antiplasmodial properties of gamhepathiopine against the blood stage of K1 P. falciparum, we highlighted that this molecule showed submicromolar antiplasmodial activity against the liver stages of P. falciparum in human hepatocytes and also that it remained active toward the blood stage of quiescent artemisinin-resistant parasites. Moreover, we showed that gamhepathiopine inhibited the formation and development of gametocytes and exflagellation of gametes and exerted *in vivo* activity in blocking transmission from infected mice to *Anopheles* mosquitos. Additional medicinal chemistry works on this novel antimalarial chemotype are now awaited to develop a new antimalarial drug candidate, focusing on improving hepatic microsomal stability.

## MATERIALS AND METHODS

### Ethics statement.

All procedures involving murine models complied with European regulations. The protocol was ethically approved by the Ministère de l’Education Nationale, de l’Enseignement Supérieur et de la Recherche (authorization number 01737.03).

The use of nonhuman primates at CEA is in accordance with the recommendations of the Weatherall report (38). Experimental procedures were conducted in strict accordance with the recommendations of the European guidelines for the care and use of laboratory animals (European directive 63/210). The protocols and the use of hepatocytes for the purpose of the work described here were approved by the Ethical Animal Committee of the CEA (permit number A 92-032-02).

### Study on the liver stage. (i) Parasite strains, sporozoite isolation, and animals.

P. falciparum (strain NF54) sporozoites were obtained from infected salivary glands of Anopheles stephensi 14 to 21 days after an infective blood meal (Department of Medical Microbiology, Radboud University Medical Center, Nijmegen, Netherlands).

P. cynomolgi (strain M) sporozoites were obtained from salivary glands of in-house-infected Anopheles stephensi mosquitoes 14 to 16 days after an infective blood meal. P. cynomolgi-infected blood was obtained from Macaca fascicularis monkeys (the natural host of P. cynomolgi) 10 to 15 days after their infection with cryopreserved blood stages of this parasite (CEA, Fontenay aux Roses, France).

P. yoelii 230-GFP-Luc sporozoites are mutant parasites expressing a green fluorescent protein (GFP)-luciferase cassette generated by the gene out marker out (GOMO) strategy (39). Sporozoites were obtained from salivary glands of in-house-infected Anopheles stephensi mosquitoes 14 to 16 days after an infective blood meal on a mouse previously infected with cryopreserved blood-stage parasites.

All infected salivary glands were removed by hand dissection, crushed in a tissue glass grinder for sporozoite isolation, and filtrated through a 40-μm filter to remove mosquito debris (cell strainer; BD Biosciences, USA). The sporozoites were counted using a disposable Glasstic microscope slide (KOVA, USA). All mice used in these experiments were female BALB/c mice (the average weight was approximately 18 g) purchased from René Janvier (Le Genest-Saint-Isle, France).

### (ii) Hepatocytes.

Primary human hepatocytes were isolated from liver segments obtained from adult patients undergoing partial hepatectomy (Service de Chirurgie Digestive, Hépato-Bilio Pancréatique, Hôpital Pitié Salpêtrière, Paris, France). Primary simian hepatocytes were isolated from liver segments collected from healthy Macaca fascicularis monkeys from CEA, Fontenay aux Roses, France. All hepatocytes were obtained using collagenase perfusion as previously described (40). Simian hepatocytes were immediately cryopreserved with a Nicool-Freezal controlled rate freezer (Air Liquide Santé, Marne la Vallée, France) and then used when needed after fast thawing at 37°C. Primary murine hepatocytes were isolated from liver segments collected from BALB/c mice. Human and murine hepatocytes were used directly after their isolation. Cells were seeded in 96-well plates (Falcon; Becton Dickinson Labware Europe, France) coated with collagen I (BD Bioscience, USA) at a density of 80,000 cells per well. Simian hepatocytes were maintained at 37°C in 5% CO_2_ in a complete William’s E medium, Gibco (Thermofisher), ref 22551-022, supplemented with 10% bovine serum fetal clone III (Hyclone), penicillin (100 U/mL), and streptomycin (100 mg/mL) (Sigma-Aldrich, USA), 5 · 10^−3^ g/liter insulin (Sigma-Aldrich, USA), and 5 · 10^−5^ M hydrocortisone (Upjohn, SERB, France) until infection with sporozoites. For human hepatocytes, the complete medium was supplemented with 2% dimethyl sulfoxide (DMSO; Sigma-Aldrich, USA) before infection to preserve their differentiation.

HepG2-CD81 cells (ATCC HB-8065 with induced expression of CD81) were cultured in 96-well plates coated with collagen I at a density of 250,000 cells/cm^2^ in Dulbecco modified Eagle medium (DMEM) supplemented with 10% fetal calf serum and antibiotics (41).

### (iii) *In vitro* cell viability assay toward primary hepatocytes and HepG2-CD81 and HepG2-HB-8065 cell lines.

The toxicity of M1 toward primary hepatocytes and HepG2-CD81 cells was evaluated through the MTT {[3-(4,5-dimethyl-2-thiazolyl)-2,5-diphenyl-2H-tetrazolium bromide]} test (42). Cells were plated (80,000 cells/well) and incubated with different concentrations of each drug in complete medium. The protocol and schedule for changing the medium were the same as for the drug assay: at 3 h postinitiation and then daily until day 2 for HepG2-CD81 cells and murine hepatocytes, and until day 5 and 8 for human and simian hepatocytes, respectively. Cultures were washed twice with phosphate-buffered saline (PBS) to eliminate complete medium. A solution of 0.5 mg/mL in Williams’s medium was added (100 μL/well) and left on the cells for 4 h at 37°C. Then, MTT was replaced by a mixture of DMSO-ethanol (1:1) to dissolve formazan crystals and the absorbance was read at 540 nm on a FlexStation plate reader.

The HepG2 cell line (HB-8065; ATCC) was maintained at 37°C, 5% CO_2_, with 90% humidity in minimum essential medium (MEM) supplemented with 10% fetal bovine serum, 1% l-glutamine (200 mM), penicillin (100 U/ml), and streptomycin (100 μg/ml) (complete MEM medium). The evaluation of the cytotoxicity of the tested molecules on the HepG2 cell line was performed according to the method of Mosman (42) with slight modifications. Briefly, 5 · 10^3^ cells in 100 μl of complete medium was inoculated into each well of 96-well plates and incubated at 37°C in a humidified 5% CO_2_ atmosphere. After 24 h of incubation, to obtain adherent cells, 100-μl amounts of medium with various product concentrations dissolved in DMSO (final concentration less than 0.5% [vol/vol]) were added and the plates were incubated for 72 h at 37°C. Triplicate assays were performed for each sample. Each well was then examined under a microscope to detect possible precipitate formation before the medium was aspirated from the wells. An amount of 100 μl of MTT solution (0.5 mg/ml in medium without FBS) was then added to each well. Cells were incubated for 2 h at 37°C to allow MTT oxidation by mitochondrial dehydrogenase in the viable cells. After this time, the MTT solution was removed and DMSO (100 μl) was added to dissolve the resulting blue formazan crystals. Plates were shaken vigorously (700 rpm) for 10 min. The absorbance was measured at 570 nm with 630 nm as the reference wavelength using a Tecan Infinite F-200 microplate reader. DMSO was used as a blank and doxorubicin as a positive control. Cell viability was calculated as the percentage compared to the control (cells incubated without compound). The 50% cytotoxic concentration (CC_50_) was determined by nonlinear regression analysis processed on the dose-response curve by using TableCurve 2D software version 5.0. CC_50_ values represent the mean values calculated from three independent experiments.

### (iv) Assessment of liver-stage development *in vitro*.

Hepatocytes were seeded into collagen-coated black 96-well plates and maintained at 37°C in 5% CO_2_ in complete medium. On the day of infection, 3 · 10^4^ sporozoites of P. falciparum, P. yoelii, and P. cynomolgi were added to their respective host cell cultures (human, murine, and simian). The infected culture plates were centrifuged for 10 min at 900 × *g*, allowing fast parasite sedimentation, and further incubated with serial dilutions of M1. Culture medium containing the appropriate drug concentration was refreshed daily, and cells were fixed at 48 h postinfection (p.i.) for infections with P. yoelii, 6 days p.i. for P. falciparum, or 8 days p.i. for P. cynomolgi. For the *in vitro* sporozoite invasion experiments, P. falciparum sporozoites were preincubated with 1, 10, and 100 μM M1 for 1 h at room temperature before hepatocyte infections. Three hours later, after sporozoite penetration into hepatocytes, cultures were washed and then further incubated with or without the presence of the drug during 6 days. DMSO was used as a negative control.

### (v) Parasite counting and schizont size determination using high-content imaging.

The number, size, and shape of the parasites were determined using a CellInsight high-content screening platform equipped with Studio HCS software (ThermoFisher Scientific). The parasite immunostaining was done using an anti-*Pf*HSP70 antibody raised in mice and a secondary anti-mouse antibody coupled to Alexa Fluor 488 (43). Host cell and parasite nuclei were labeled with DAPI (4′,6-diamidino-2-phenylindole). Thirty-seven images, representing more than 95% of the total bottom surface area of a culture well in 96-well plates, were captured for analysis. The custom script used for counting and measuring parasites first required identification of the objects based on a fluorescence intensity threshold. The object identity was then further validated based on size and morphological (shape) criteria. The presence of DAPI-associated fluorescence in the selected objects allowed their final selection and the rejection of false positives. EC_50_s are the drug concentrations at which a 50% reduction of the number of exoerythrocytic forms (EEFs) was observed compared to the numbers in control cultures. EC_50_ (estimated with ICEstimators software version 1.2) is the drug concentration at which a 50% reduction of the number of EEFs was observed compared to the numbers in control cultures (44). The parasitic size reduction was calculated on the average object area using the total surface area of each selected object (μm^2^).

### (vi) *In vivo* assessment of the effect toward the liver stage.

Five mice per treatment group were used. Experimental groups were treated with 50 and 100 mg/kg of M1 intraperitoneally (i.p.). Untreated infected mice and noninfected mice (infection controls), as well as primaquine-treated (50 mg/kg) mice (control drug), were also included. The drugs were administered on days −1, 0, and +1, and mice were challenged on day zero by retroorbital injection of P. yoelii (GFP-luc strain) sporozoites (10,000 per mouse). *In vivo* bioluminescence imaging was performed 44 h postinfection on day 2, to assess liver-stage development. When parasites were detected in the liver, treatment was continued until day 5 and *in vivo* imaging was performed on days 3 and 6, to evaluate a prepatent period for the blood-stage parasites in mice after the sporozoite inoculation. *In vivo* imaging and blood smears were performed to monitor blood-stage development using methods described previously (45). This experiment was performed twice.

IVIS Spectrum (Caliper Life Science, Hanover, MD) was used to capture bioluminescence signals. Prior to analysis, the mice were injected i.p. with d-luciferin (100 mg/kg), anesthetized with isoflurane, and imaged 10 min postinjection. Images were analyzed using the Living Image 3.0 software (Caliper Life Sciences, Hanover, MD).

In order to study the *in vivo* efficacy of M1 without any hepatic CYP metabolism, we conducted an experiment using 1-aminobenzotriazole (ABT), a nonselective inhibitor of cytochrome P450 enzymes (34, 35). ABT was administered to mice i.p. 1 h before each treatment with 50 mg/kg gamhepathiopine for 3 days starting from the day before sporozoite inoculation.

### *In vivo* transmission-blocking activity assays.

Experimental mice were infected by i.p. inoculations of 10^7^ erythrocytes parasitized with P. yoelii GOMO 15 to test the effect of M1 following two different protocols of drug treatment. Infected mice were randomly allocated into 6 groups (5 mice per group) corresponding to the 3 conditions (control [solvent], M1 50 mg/kg, and PQ 50 mg/kg) in both drug administration protocols. For the first protocol, mice were treated daily from day zero to day 3, while for the second protocol, treatment was administered only once, 4 days after the infection and 2 h before mosquito feeding (37). At day 3 p.i., the presence and maturity of gametocytes was checked by microscopic examination of Giemsa-stained thin blood films and live-blood observation to assess microgamete exflagellation density. Mice were then anesthetized and placed according to the treatment on the top of individual cages containing 100 glucose-starved Anopheles stephensi female mosquitoes, which were allowed to feed for 1 h. Unfed mosquitoes were removed from the cages. Seven days after the infectious blood meal, 30 mosquitoes were dissected and their midguts examined using a phase-contrast microscope (400×) to assess the presence of oocysts. Two weeks after the blood meal, the salivary glands of 30 mosquitos were dissected to estimate the average number of sporozoites per mosquito in each group.

### P. falciparum gametocyte induction and *in vitro* drug susceptibility assays.

To produce gametocytes, P. falciparum strain NF54 was cultured *in vitro* as described by Trager and Jensen (46) with minor modifications. Briefly, parasites were maintained in human type O-positive red blood cells (RBC) at 5% hematocrit (HCT) in RPMI 1640 medium supplemented with 10% heat-inactivated human serum, 10 mM hypoxanthine, 20 μg/ml gentamicin, The cultures were maintained at 37°C in a standard gas mixture of 5% O_2_, 5% CO_2_, 90% N_2_. Synchronous production of gametocytes was achieved as previously described (47). Briefly, asexual parasite cultures were synchronized by purifying schizonts with MACS cell separation columns (Miltenyi Biotec) and using them to reinvade erythrocytes. When parasitemia reached 10 to 15% rings, culture medium was supplemented with 50 mM *N-*acetylglucosamine (NAG; Sigma-Aldrich) for 5 days in order to clear residual asexual parasites and obtain a virtually pure gametocyte culture.

To assess the effect of gamhepathiopine on the induction of gametocytes, M1 (10 μM) was added at the same moment with NAG. For gametocyte development, gamhepathiopine was added 2 days after the addition of NAG. Medium replacement was performed every day. All cultures were prepared in parallel with controls corresponding to cultures that were treated with 0.1% DMSO. The percentages of each stage of gametocytes in cultures were assessed by microscopy of Giemsa-stained thin blood smears.

### Exflagellation-blocking assays.

Exflagellation-blocking assays were performed according to published protocols (48, 49). Assays were performed using gametocyte cultures providing high-enough numbers of exflagellation centers for meaningful measurement (>30 centers of exflagellation in 50 microscope fields at 400× magnification).

To show the suitability of field isolates for exflagellation-blocking assays, the activity of 10 μM M1 was evaluated. Assays were carried out in 6-well plates containing 400 μl of gametocyte culture medium with 10 μM M1 dissolved in DMSO. Then, 100 μl of stage V mature gametocytes at 2% HCT were dispensed into each assay well. The 6-well plate was incubated at 37°C. After treatment with M1 during 2 h or 24 h, exflagellation was induced by decreasing the temperature to 25°C and replacing culture medium with AB serum. The numbers of exflagellation centers were recorded between 10 and 30 min after induction and compared to the numbers in the control (DMSO treatment). Activity was expressed as the percentage of exflagellation inhibition compared to the control.

### Statistical analysis for the studies regarding the hepatic stage, gametocytes, and sporozoites.

Excel 2007 (Microsoft Office) and GraphPad Prism 6 statistical software (GraphPad Software, San Diego, CA, USA) were used for data analysis. All values were expressed as means and standard deviations (SD). A *P* value of 0.05 or less was considered statistically significant.

### *In vitro* chemosensitivity assay regarding the blood stage of P. falciparum.

In this study, a K1 culture-adapted P. falciparum strain resistant to chloroquine, pyrimethamine, and proguanil was used in an *in vitro* culture. Maintenance in continuous culture was done as described previously by Trager and Jensen (46). Cultures were maintained in fresh A+ human erythrocytes (EFS [French Blood Bank]) at 2.5% hematocrit in complete medium (RPMI 1640 with 25 mM HEPES, 25 mM NaHCO_3_, 10% A+ human serum) at 37°C under a reduced-O_2_ atmosphere (gas mixture of 10% O_2_, 5% CO_2_, and 85% N_2_). Parasitemia was maintained daily between 1% and 3%. The P. falciparum drug susceptibility test was carried out by comparing quantities of DNA in treated and control cultures of parasites in human erythrocytes according to a SYBR green I fluorescence-based method (50) using a 96-well fluorescence plate reader. Compounds, previously dissolved in DMSO (final concentration, less than 0.5% vol/vol), were incubated in a total assay volume of 200 μl of synchronized culture suspension (2% hematocrit and 0.4% parasitemia) for 72 h in a humidified atmosphere (85% N_2_, 10% O_2_, and 5% CO_2_) at 37°C in 96-well flat-bottom plates. Duplicate assays were performed for each sample. After incubation, plates were frozen at −20°C for 24 h. Then, the frozen plates were thawed for 1 h at 37°C. Fifteen microliters of each sample was transferred to 96-well flat-bottom black plates (Greiner Bio-one) already containing 15 μl of the SYBR green I lysis buffer (2× SYBR green I, 20 mM Tris base, pH 7.5, 20 mM EDTA, 0.008% [wt/vol] saponin, 0.08% [wt/vol] Triton X-100). A negative control, treated by solvents (DMSO or H_2_O), and positive controls (atovaquone, chloroquine, and doxycycline) were added to each set of experiments. Plates were incubated for 15 min at 37°C and then read on a Tecan Infinite F-200 spectrophotometer with excitation and emission wavelengths at 485 and 535 nm, respectively. The concentrations of compounds required to induce a 50% decrease in parasite growth (EC_50_K1) were calculated by nonlinear regression analysis processed on the dose-response curve by using TableCurve 2D version 5.0. EC_50_K1 values represent the mean values calculated from three independent experiments.

### Evaluation of M1 activity in the framework of resistance to artemisinin. (i) Parasite culture.

The Plasmodium falciparum artemisinin-resistant strain F32-ART5 and its twin artemisinin-sensitive line F32-TEM (30, 31) were cultivated at 37°C with 5% CO_2_ in human red blood cells (EFS [French Blood Bank]) diluted to 2% hematocrit in RPMI 1640 medium supplemented with 5% human serum (EFS) as previously reported (47).

### (ii) Standard *in vitro* chemosensitivity assay.

*In vitro* susceptibility of P. falciparum parasites was determined by using the standard hypoxanthine uptake inhibition assay as described previously (51) with some modifications (52). After d-sorbitol treatment, parasites synchronized at the ring stage were exposed for 48 h, with [^3^H]hypoxanthine (Perkin Elmer, France), to a range of concentrations of the different tested compounds. [^3^H]hypoxanthine incorporation was then measured with a β-counter, and EC_50_s (concentrations of drugs that inhibited 50% of the parasite growth) were determined in concentration versus percent inhibition curves. Each result represents 3 independent experiments.

### (iii) Recrudescence assay.

A recrudescence assay was performed to evaluate the ability of the F32-ART5 parasite line to survive drug exposure in comparison to that of the F32-TEM parasite line (30, 31) in order to evaluate the risk of cross-resistance between artemisinin and M1. d-Sorbitol-synchronized ring-stage parasites at 3% parasitemia (2% hematocrit) were exposed to the drug for 48 h. After drug exposure, parasite cultures were washed with RPMI 1640 medium and replaced in normal drug-free culture conditions. The time to parasite recrudescence was determined as the time required to reach initial parasitemia. If no parasites were observed in the following 30 days, the culture was considered not recrudescent (30, 31). Each experiment, performed in parallel for F32-ART5 and F32-TEM cultivated under the same conditions to generate paired results, was carried out at least three times independently and data statistically analyzed. The tested doses corresponded to 10- to 50-fold the EC_50_s (31).

### (iv) QSA.

Chemosensitivity evaluation of artemisinin-resistant parasites at the quiescent stage was performed on the F32-ART5 strain using the quiescent-stage survival assay (QSA) (33). d-Sorbitol-synchronized ring-stage parasites at 3% parasitemia (2% hematocrit) were first exposed to 3 μM artemisinin for 6 h to induce quiescence of artemisinin-resistant parasites. Then, quiescent parasites were exposed or not (as a control condition) to 2.5 μM M1 for 24 h. After drug exposure, parasite cultures were washed with RPMI 1640 medium and replaced in normal drug-free culture conditions. The time to parasite recrudescence was determined as the time required to reach initial parasitemia. If no parasites were observed in the following 30 days, the culture was considered not recrudescent. In each experiment, results obtained with the condition “artemisinin 3 μM 6 h/artemisinin 3 μM 24 h” were compared to those obtained with the condition “artemisinin 3 μM 6 h/(artemisinin 3 μM + M1 2.5 μM) 24 h” in order to determine the delay in recrudescence time. This delay is representative of the capacity of the tested compound to be active on quiescent parasites, and it is assumed that a 6-day threshold is necessary to classify a compound as active on quiescent parasites (33). Each experiment was carried out three times independently, and data statistically analyzed. The same experiment was done once with chloroquine (CQ) at 200 nM as a negative control because, despite its activity on proliferating *Plasmodium* parasites, CQ fails to kill quiescent parasites due to its therapeutic target, which is not implemented in quiescent parasites (33).

### (v) Data analysis.

A statistical analysis (log-rank test) was carried out on data from the recrudescence assay and the QSA using a Kaplan-Meier survival analysis considering censored data. The final event was defined as the time necessary for cultures to reach initial parasitemia. Observations were considered censored if no recrudescence was observed within the experiment time frame (30 days).

### Microsomal stability and metabolite identification.

The tested product and propranolol, used as a reference, were incubated in duplicate (reaction mixture volume of 0.5 ml) with female mouse microsomes (CD-1, 20 mg/ml; BD Gentest) at 37°C in a 50 mM phosphate buffer, pH 7.4, in the presence of MgCl_2_ (5 mM), NADP (1 mM), glucose-6-phosphate dehydrogenase (G6PD) (0.4 U/ml), and glucose-6-phosphate (5 mM). For the estimation of the intrinsic clearance, 50-μl aliquots were collected at 0, 5, 10, 20, 30, and 40 min and the reaction was stopped with 4 volumes of acetonitrile (ACN) containing the internal standard. After centrifugation at 10,000 × *g* for 10 min at 4°C, the supernatants were conserved at 4°C for immediate analysis or placed at −80°C in case of postponement of the analysis. Controls (time zero and final time point) in triplicate were prepared by incubation of the internal standard with microsomes denatured by acetonitrile. The LC-MS system used for this study was a Waters Acquity I-Class/Xevo TQD equipped with a Waters Acquity BEH C_18_ column, 50 by 2.1 mm, 1.7 μm. The mobile phases were ammonium acetate 10 mM (mobile phase A) and acetonitrile with 0.1% formic acid (mobile phase B). The injection volume was 1 μl, and the flow rate was 600 μl/min. The chromatographic analysis, with a total duration of 4 min, was made with the following gradient: 0 < *t* < 0.2 min, 2% mobile phase B; 0.2 < *t* < 2 min, linear increase to 98% mobile phase B; 2 < *t* < 2.5 min, 98% mobile phase B; 2.5 < *t* < 2.6 min, linear decrease to 2% mobile phase B; 2.6 < *t* < 4 min, 2% mobile phase B. The quantification of each compound was obtained by converting the average of the ratios of the analyte/internal standard surfaces to the percentage of consumed product. The ratio of the control at *t*0 corresponded to 0% of product consumed. The calculation of the half-life (*t*_1/2_) of each compound in the presence of microsomes was done according to the equation *t*_1/2_ = (ln_2_)/*k*, where *k* is the first-order degradation constant (the slope of the logarithm of compound concentration versus incubation time). The intrinsic clearance *in vitro* (Cl_int_, expressed in μl/min/mg) was calculated according to the equation Cl_int_ = (dose/AUC_∞_)/[microsomes], where dose is the initial concentration of product in the sample, AUC_∞_ is the area under the concentration-time curve extrapolated to infinity, and [microsomes] is the microsome concentration expressed in mg/μl.

Gamhepathiopine (0.5 ml of a 37.5 μM solution in water/methanol 99:1) was incubated with female mouse microsomes (CD-1, 20 mg/ml; BD Gentest) at 37°C in a 50 mM phosphate buffer, pH 7.4, in the presence of MgCl_2_ (5 mM), NADP (1 mM), glucose-6-phosphate dehydrogenase (0.4 U/ml) and glucose-6-phosphate (5 mM). The reaction was stopped by adding 2 ml acetonitrile at *t* = 0, *t* = 2 min, *t* = 5 min, and *t* = 15 min. After centrifugation (12,000 rpm for 10 min at 4°C), supernatants were evaporated *in vacuo* and reconstituted in a methanol/acetonitrile mixture (1:1) before analysis. Controls (*t* = 0, *t* = 2 min, *t* = 5 min, and *t* = 15 min) were prepared by incubation of gamhepathiopine with microsomes denatured by acetonitrile. The analytical methods used for metabolite identification were LC-time of flight (TOF) MS (Waters LCT Premier XE) and ultra-high-performance liquid chromatography (UHPLC)-triple quadrupole MS (Waters Acquity I-Class/Xevo TQD). Among the identified metabolites of M1, alcohol **7**, aldehyde **8**, and carboxylic acid **10** were synthesized and studied under the same chromatography and mass spectrometry analysis conditions for validation purposes.
