# Supplementary material for: A New Thienopyrimidinone Chemotype Shows Multistage Activity against Plasmodium falciparum, Including Artemisinin-Resistant Parasites
Source: Microbiol Spectr. 2021 Sep 29;9(2):e00274-21. doi: 10.1128/Spectrum.00274-21 (PMC8557901; doi:10.1128/Spectrum.00274-21)
Supplement: SUPPLEMENTAL FILE 1 — Supplemental material. Download SPECTRUM00274-21_Supp_1_seq4.docx, DOCX file, 0.5 MB. [file spectrum00274-21_supp_1_seq4.docx]

Supporting information

A new thienopyrimidinone chemotype shows multi-stage activity against Plasmodium falciparum, including artemisinin-resistant parasites

Henriette Bosson-Vanga, Nicolas Primas, Jean-François Franetich, Catherine Lavazec, Lina Gomez, Kutub Ashraf, Maurel Tefit, Valérie Soulard, Nathalie Dereuddre-Bosquet, Roger Le Grand, Mélanie Donnette, Nadia Amazougaghene, Shahin Tajeri, Peggy Suzanne, Sylvain Rault, Patrice Vanelle, Sébastien Hutter, Anita Cohen, Georges Snounou, Pierre Roques, Nadine Azas, Vincent Lisowski, Michel Nguyen, Lucie Paloque, Françoise Benoit-Vical, Pierre Verhaeghe, Dominique Mazier

T**able of content**

Figure S1………………………………………………………………………………………………………..p 2

Figure S2………………………………………………………………………………………………………..p 2

Figure S3………………………………………………………………………………………………………..p 3

hERG binding assay………..………………………………………………………………………………….p 3

Cytotoxicity on MDCK renal cell line………………………………………………………………………….p 3

Drugs and chemicals…………………………………………………………………………………………...p 4

Synthesis of compounds 2-10, including new synthesis pathway for Gamhepathiopine (M1 or 6)……p 4

Figure S4………………………………………………………………………………………………………..p 6

References……………………………………………………………………………………………………...p 6

**Figure S1.** New chemical entities displaying multi-stage antimalarial activity which have reached clinical studies.

**Figure S2.** Protocol of *in vitro* gametocytocidal activity assays (NAG = *N*-acetylglucosamine). Gametocytes stages: I, II, III, IV and V. In blue: M1 effect on gametocyte induction: M1 (10 µM) added at D0 on asexual stage before induction with NAG (control= NAG+ DMSO). In green: M1 effect on gametocyte development: M1 (10 µM) added at D3 after induction (Control= DMSO). In red: M1 effect on exflagellation of mature male gametocytes on D10 (Control DMSO)


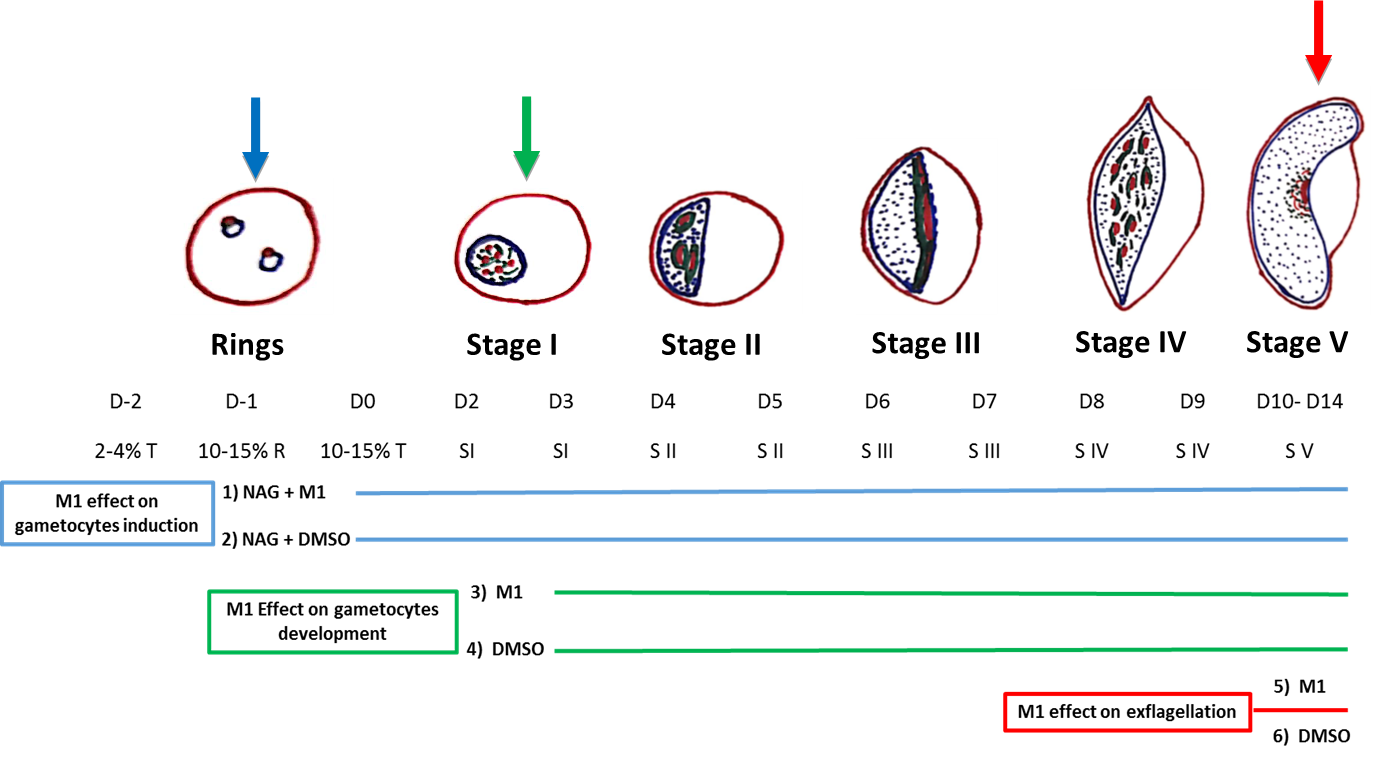


**Figure S3.** hERG channel binding concentration response curves determined for M1and a known hERG channel blocker (E-4031), generated by the predictor® hERG fluorescence polarization assay kit.


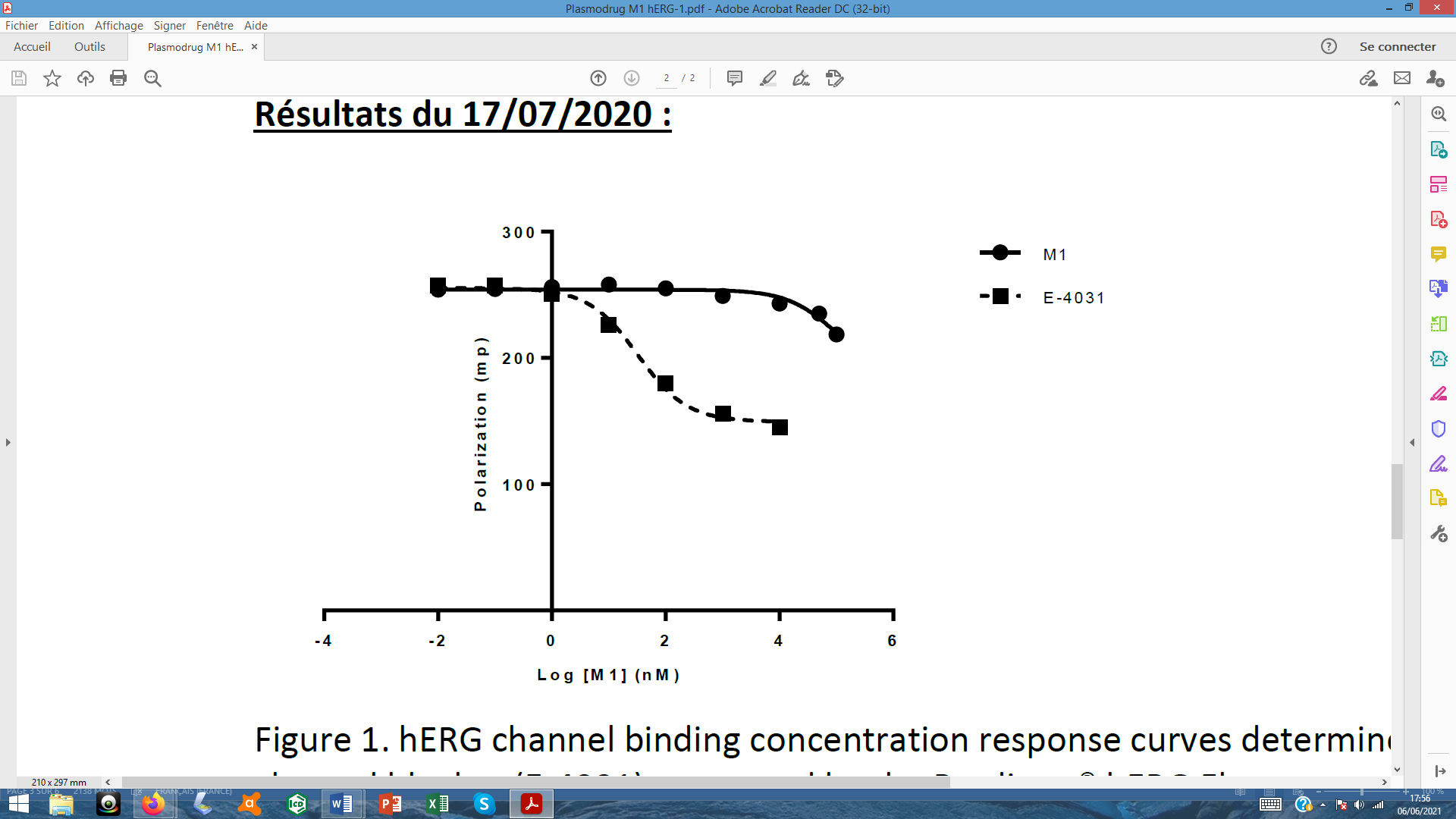


**hERG binding assay**

hERG channel binding was determined with the PredictorTM hERG Fluorescence Polarization Assay (PV5665 ThermoFischer). Briefly, reagents were thawed, and were mixed by pipetting 20x with PredictorTM hERG membranes. The fluorescent tracer was diluted at 4 nM in the PredictorTM hERG buffer. The tested compound was dissolved in DMSO at 10 mM, and was then diluted from 0.4 µM to 0.04 nM in the PredictorTM hERG buffer. The assay was performed in a 96-well Greiner microplate (675093 Dutscher). 25 µL of each concentration of M1 were dispensed into the plate. Then, 50 µL of the PredictorTM hERG membranes and 25 µL of the fluorescent tracer 4 nM were added. The plate was incubated at room temperature for 4 h. The fluorescence polarization measurements were made using a Synergy 2 microplate reader (Biotek Instrument, Colmar, France). Both parallel and perpendicular fluorescence were measured using 530/25 nm excitation and 590/35 nm emission filters with a 570 nm dichroic mirror. The gain was fixed at 110. A known hERG channel blocker (E-4031), provided by the kit, was assayed as positive reference. Both E-4031 and M1 were tested at final concentrations ranging from 0.01 nM to 100 µM. IC_50_ values were determined graphically by plotting the % inhibition versus the logarithm of 9 M1 concentrations in the assay solution using the GraphPad Prism software (version 6.01, GraphPad Software, La Jolla, CA, USA).

**Cytotoxicity on the MDCK renal cell line**

MDCK cell line (Madin-Darby Canine Kidney cells) was maintained at 37 °C, 5% CO_2_ with 90% humidity in DMEM supplemented with 5% fetal bovine serum, 1% L-glutamine (200 mM), penicillin (100 U/mL) and streptomycin (100 μg/mL) (complete medium). The evaluation of Gamhepathiopine cytotoxicity on this cell line was performed according to the method of Mosman with slight modifications. Briefly, 3.10^4^ cells in 100 μL of complete medium were inoculated into each well of 96-well plates and incubated at 37 °C in a humidified 5% CO_2_. After 24 h incubation, to obtain adherent cells, 100 μL of medium with various concentrations of Gamhepathipoine dissolved in DMSO were added and refreshed on a daily basis for 72 h at 37 °C. Each sample was tested on 4 technical replicates. The MTT solution (100µL/well, 0.5 mg/mL in medium without FBS) was then added to each well. Cells were incubated for 4 h at 37 °C. The MTT solution was then removed and a mixture of DMSO/Ethanol (1:1 ; 100 μL/well) was added to dissolve the resulting blue formazan crystals. The absorbance was measured at 540 nm on a Flex Station Microplate Reader. The 50% cytotoxic concentration (CC_50_) was determined by non-linear regression analysis processed on the dose–response curve by using ICEstimator (http://www.antimalarial-icestimator.net/). The resulting CC_50_ obtained was 39.5 µM (+/-4.4) with a 95% confidence interval.

**Drugs and chemicals**

For the *in vitro* assay, tested compounds were dissolved in dimethyl sulfoxide (DMSO) to prepare stock solutions. In the in vivo studies, the compound injected intraperitoneally was dissolved in 10% DMSO – 0.5% TWEEN and given at 5, 10 and 50 mg/kg. Primaquine was obtained from Sigma-Aldrich, USA. Stocks solutions were prepared at 10 mg/mL in distilled water. D-Luciferin, a substrate for luciferase, was provided by OZ Biosciences and was dissolved in 1X Phosphate buffered saline (PBS, Gibco) and injected intraperitoneally at 100 mg/kg. 1-Aminobenzotriazole (ABT) was purchased from Sigma-Aldrich, USA and used *in vivo* as a non-selective inhibitor of cytochrome P450 enzymes. Stocks solutions were prepared at 10 mg/mL in distilled water.

Chloroquine diphosphate salt was purchased from Sigma-Aldrich, USA. Stocks solutions were prepared at 10 or 40 mM in ultrapure water. Doxycycline hyclate was purchased from Sigma-Aldrich, USA. Stocks solutions were prepared at 50 mM in ultrapure water. Doxorubicine was purchased from Sigma-Aldrich, USA. Stocks solutions were prepared at 25 mM in ultrapure water. Atovaquone was purchased from Sigma – Aldrich, USA. Stock solutions were prepared in DMSO.

**Synthesis of compounds 2-10, including new synthesis pathway for Gamhepathiopine (M1 or 6)**

Reagents were purchased from commercial sources or prepared following standard literature procedures. Unless otherwise noted, reactions were carried out under atmosphere. Concentration under vacuum refers to the removal of solvent on a Buchi Rotary Evaporator. The microwave reactions were performed using a Biotage Initiator Microwave oven using sealed vials; temperatures were measured with an IR-sensor and reaction times given as hold times. Proton magnetic resonance (^1^H NMR) spectra were recorded on a Bruker AV 250 spectrometer or a BRUKER Avance III nanobay 400. All spectra were determined in the indicated solvents. Although chemical shifts are reported in ppm downfield of tetramethylsilane, they are referenced to the residual proton peak of the respective solvent peak for ^1^H NMR. Inter-proton coupling constants are reported in Hertz (Hz). LCMS spectra were obtained using a Thermo Scientific Accela High Speed LC System® coupled with a single quadrupole mass spectrometer Thermo MSQ Plus®. The RP-HPLC column used is a Thermo Hypersil Gold® 50 × 2.1 mm (C_18_ bounded), with particles of 1.9 µm diameter. The volume of sample injected on the column was 1 µL. The chromatographic analysis, total duration of 8 min, is made with the gradient of following solvents: t = 0 min, water/methanol 50/50; 0 < t < 4 min, linear increase in the proportion of methanol to a ratio water/methanol 5/95; 4 < t < 6 min, water/methanol 5/95; 6 < t < 7 min, linear decrease in the proportion of methanol to return to a ratio 50/50 water/methanol; 6 < t < 7 min, water/methanol 50/50. The water used was buffered with 5 mM ammonium acetate. HRMS spectra were recorded on QStar Elite (Applied Biosystems SCIEX) spectrometer. PEG was the matrix for HRMS. The experimental exact mass was given for the ion which has the maximum isotopic abundance. The structures of all synthesized molecules (**2-10**) are presented in **Figure S2**.

**Methyl 3-(2,2,2-trichloroacetamido)thiophene-2-carboxylate 2 (38)**

Methyl 3-aminothiophene-2-carboxylate 1 (3.54 g, 22.52 mmol) was poured in dry THF (30 mL) under inert atmosphere at 0 °C and triethylamine was added (3.13 mL, 22.52 mmol). Then, trichloroacetyl chloride (2.51 mL, 22.52 mmol) was added dropwise and the temperature was kept at 0 °C 20 min after the end of the addition. The mixture was stirred 2 h at RT. After the completion of the reaction, water was added and the volatiles were removed under vacuum. The resulting mixture was extracted twice with EtOAc. The organic layer was rinsed with brine, dried with anhydrous sodium sulfate and concentrated under vacuum, affording the desired product **2** as a yellowish powder (6.60 g, 97%).

MP: 91 °C. ^1^H NMR (CDCl3, 250 MHz) *δ* 11.60 (bs, 1H, NH), 8.08 (d, J = 5.5Hz, 1H), 7.57 (d, J = 5.5 Hz, 1H), 3.94 (s, 3H). ^13^C NMR (CDCl3, 63 MHz) *δ* 164.8, 159.3, 142.7, 132.3, 121.9, 113.2, 92.5, 52.6.

**Methyl 5-bromo-3-(2,2,2-trichloroacetamido)thiophene-2-carboxylate 3 (1)**

To a three-neck round-bottom flask was dissolved, with the assistance of ultrasound, methyl 3-(2,2,2-trichloroacetamido)thiophene-2-carboxylate 2 (6.60 g, 21.81 mmol) in glacial acetic acid (66 mL). Bromine (3.37 mL, 65.44 mmol) was added dropwise with a dropping funnel at RT. Then, the mixture was heated at 70 °C for 10 h. After cooling down, the excess of bromine was neutralized with sodium thiosulfate and the pH was adjusted to 7 with Na_2_CO_3_. Extraction was made with CH_2_Cl_2_, the combined organic layers were washed with water, dried with anhydrous sodium sulfate and concentrated under vacuum. The resulting residue was purified by column chromatography (eluent: petroleum ether 99 /diethyl ether 1) affording the desired compound **3** as a white solid (3.90 g, 47%).

MP: 99 °C. ^1^H NMR (CDCl3, 250 MHz) *δ* 11.56 (bs, 1H, NH), 8.12 (s, 1H), 3.92 (s, CH3). ^13^C NMR (CDCl3, 63 MHz) *δ* 163.7, 159.2, 142.2, 125.0, 121.7, 114.0, 91.2, 52.8.

**Methyl 3-amino-5-bromothiophene-2-carboxylate 4 (1,2)**

Methyl 5-bromo-3-(2,2,2-trichloroacetamido)thiophene-2-carboxylate 3 (3.38 g, 8.86 mmol) and potassium carbonate (3.67 g, 26.58 mmol) were mixed in 35 mL of methanol. The mixture was stirred at RT upon completion of the reaction (24 h). The volatiles were removed under vacuum and the residue was partitioned between water and EtOAc. The organic layer was dried with anhydrous sodium sulfate and concentrated under vacuum. The resulting residue was purified by column chromatography (eluent: petroleum ether 9 /EtOAc 1) affording the desired compound **4** as an off-white solid (1.59 g, 75%).

MP: 86 °C. ^1^H NMR (CDCl_3_, 250 MHz) *δ* 6.58 (s, 1H), 4.76 (bs, 2H, NH2), 3.80 (s, 1H). ^13^C NMR (CDCl_3_, 63 MHz) *δ* 164.0, 153.5, 123.1, 120.9, 102.2, 51.5.

**6-Bromo-2-(*tert*-butylamino)thieno[3,2-d]pyrimidin-4(3*H*)-one 5**

A mixture of methyl 3-amino-5-bromothiophene-2-carboxylate **4** (2.0 g, 8.5 mmol) and of ethoxycarbonyl isothiocyanate (1 mL, 8.5 mmol) in dimethylformamide (40 mL) was stirred during 1 h at RT. Then, triethylamine (3.54 mL, 25 mmol), *tert*-butylamine (1.78 mL, 17 mmol) and 1-ethyl-3-(3-dimethylaminopropyl)carbodiimide hydrochloride (1.38 g , 0.0089 mol) were successively added to the mixture. The reaction was stirred during 16 h at RT and then heated to 160 °C in a sealed vial under microwave irradiation. After cooling down and removal of the volatiles, the resulting residue was purified by column chromatography (eluent: petroleum ether/ethyl acetate). The obtained powder was recrystallized in acetonitrile. The desired product **5** was obtained as a white powder (1.31 g, 51%).

HRMS (m/z): 301.9954 (M+H) (Calcd. for C_10_H_13_BrN_3_OS^+^ 301.9957). MP: 225 °C. ^1^H NMR (250 MHz, CDCl_3_) *δ* 7.10 (s, 1H), 6.13 (bs, 1H, NH), 1.48 (s, 9H).

^13^C NMR (63 MHz, CDCl_3_) *δ* 159.1, 152.0, 127.5, 124.7, 113.4, 52.3, 29.0.

**General procedure for the synthesis of 6-aryl-2-(*tert*-butylamino)thieno[3,2-*d*]pyrimidin-4-one derivatives 6-8**

In a microwave vial, 6-bromo-2-(*tert*-butylamino)thieno[3,2-*d*]pyrimidin-4(3*H*)-one **5** (1 equiv), PdCl_2_(PPh_3_)_4_ (5% mol), appropriate arylboronic acid (1.2 to 1.5 equiv), potassium carbonate 1 M in water (2.2 equiv) and dioxane (3 mL/mmol of **5**). The vial was degassed, filled with argon and sealed. The vial was heated under microwave activation at 120 °C for 1 h. The resulting mixture was partitioned between ethyl acetate and water. The organic layer was washed with brine, dried with anhydrous sodium sulfate and concentrated under vacuum. The residue was purified by column chromatography (eluent: petroleum ether/ethyl acetate) and washed with acetonitrile affording the desired compound.

**2-(*tert*-Butylamino)-6-(*p*-tolyl)thieno[3,2-*d*]pyrimidin-4(3*H*)-one 6 = Gamhepathiopine = M1 (3)**

Following the general procedure starting from **5** (0.4 g, 1.3 mmol) and *p*-tolylboronic acid (0.270 g, 2 mmol), the desired compound **6** was obtained as a white solid (0.167 g, 66%).

HRMS (m/z): 314.1323 (M+H) (Calcd. for C_17_H_20_N_3_OS^+^ 314.1322). MP > 260 °C. ^1^H NMR (250 MHz, DMSO-d6) *δ* 10.50 (bs, 1H), 7.67 (d, J = 8.1 Hz, 2H), 7.43 (s, 1H), 7.24 (d, J = 8.1 Hz, 2H), 6.07 (bs, 1H), 2.32 (s, 3H), 1.40 (s, 9H).

^13^C NMR (63 MHz, DMSO-d6) *δ* 160.7, 158.0, 152.7, 150.7, 139.5, 130.7, 130.2, 126.2, 120.4, 111.5, 51.4, 29.1, 21.3.

**2-(*tert*-Butylamino)-6-(4-(hydroxymethyl)phenyl)thieno[3,2-*d*]pyrimidin-4(3H)-one 7**

Following the general procedure starting from **5** (0.39 g, 1.3 mmol) and 4-(hydroxymethyl)phenylboronic acid (0.235 g, 1.5 mmol), the desired compound **7** was obtained as a white solid (0.167 g, 39%).

HRMS (m/z): 330.1271 (M+H) (Calcd. for C_17_H_20_N_3_O_2_S^+^ 330.1271). MP: 264 °C. ^1^H NMR (250 MHz, DMSO-d6) *δ* 10.47 (s, 1H), 7.76 (d, J = 7.8 Hz, 2H), 7.49 (s, 1H), 7.39 (d, J = 7.7 Hz, 2H), 6.09 (s, 1H), 5.29 (t, J = 4.6 Hz, 1H), 4.53 (d, J = 4.6 Hz, 2H), 1.42 (s, 9H).

^13^C NMR (63 MHz, DMSO-d6) *δ* 160.3, 157.6, 152.2, 150.1, 144.0; 131.4, 127.1, 125.7, 120.2, 111.2, 62.5, 51.0, 28.6.

**4-(2-(*tert*-Butylamino)-4-oxo-3,4-dihydrothieno[3,2-*d*]pyrimidin-6-yl)benzaldehyde 8**

Following the general procedure starting from **5** (0.50 g, 1.7 mmol) and 4-formylphenylboronic acid (0.298 g, 2 mmol), the desired compound **8** was obtained as a yellow solid (0.338 g, 62%).

HRMS (m/z): 328.1115 (M+H) (Calcd. for C_17_H_18_N_3_O_2_S^+^ 328.1114). MP: 296 °C. ^1^H NMR (250 MHz, DMSO-d6) *δ* 10.58 (bs, 1H), 10.03 (s, 1H), 8.05 (d, J = 8.6 Hz, 2H), 7.97 (d, J = 8.1 Hz, 2H), 7.74 (s, 1H), 6.15 (bs, 1H), 1.42 (s, 9H).^13^C NMR (63 MHz, DMSO-d6) *δ* 192.5, 160.1, 157.7, 152.3, 148.1, 138.3, 136.1, 130.4, 126.5, 122.7, 112.9, 51.1, 28.6.

**Methyl 4-(2-(*tert*-butylamino)-4-oxo-3,4-dihydrothieno[3,2-*d*]pyrimidin-6-yl)benzoate 9**

Following the general procedure starting from **5** (0.75 g, 2.5 mmol) and (4-(methoxycarbonyl)phenyl)boronic acid (0.536 g, 3 mmol), the desired compound **9** was obtained as a white solid (0.504 g, 57%).

HRMS (m/z): 358.1216 (M+H) (Calcd. for C_18_H_20_N_3_O_3_S^+^ 358.1220).

LCMS Rt = 4.21 min, (m/z): 358 (M+H). MP: 218 °C. ^1^H NMR (250 MHz, DMSO-d6) *δ* 10.63 (bs, 1H), 8.01 (d, J = 8.4 Hz, 2H), 7.95 (d, J = 8.5 Hz, 2H), 7.68 (s, 1H), 6.24 (bs, 1H), 3.87 (s, 3H), 1.41 (s, 9H).^13^C NMR (63 MHz, DMSO-d6) *δ* 165.7, 159.9,157.7, 152.4, 148.2, 137.3, 130.1, 129.8, 126.1, 122.3, 112.7, 52.4, 51.1, 28.6.

**4-(2-(*tert*-Butylamino)-4-oxo-3,4-dihydrothieno[3,2-*d*]pyrimidin-6-yl)benzoic acid 10**

To a solution of methyl 4-(2-(*tert*-butylamino)-4-oxo-3,4-dihydrothieno[3,2-*d*]pyrimidin-6-yl)benzoate **9** (0.504 mg, 1.4 mmol) in ethanol (4.5 mL) was added sodium hydroxide 1 M (6.5 mL). The mixture was refluxed 30 min. After cooling, the mixture was neutralized with HCl 1M and the resulting precipitate was filtrated, washed with cold water and then dried under vacuum with desiccant. The resulting residue was triturated in acetonitrile and filtrated, affording the desired compound **10** as a hydrochloride salt (0.402 g, 75%).

HRMS (m/z): 344.1065 (M+H) (Calcd. for C_17_H_18_N_3_O_3_S^+^ 344.1063). MP: >300 °C. ^1^H NMR (400 MHz, DMSO-d6) *δ* 7.92 (d, J = 8.3 Hz, 2H), 7.68 (d, J = 8.3 Hz, 2H), 7.39 (s, 1H), 1.44 (s, 9H).^13^C NMR (101 MHz, DMSO-d6) *δ* 169.2, 162.3, 160.4, 156.6, 147.8, 140.8, 133.7, 129.7, 124.5, 120.1, 111.2, 50.2, 29.0.

**Figure S4.** Novel synthesis pathway for the preparation of Gamhepathiopine (M1 = **6**) and some of its metabolites (**7**, **8** and **10**).

**References**

1. S. Zeng, W. Hu, G. Zhang, L. Zeng, Thienopyrimidinone Compound or Pharmaceutically-Acceptable Salt Thereof and Preparation Method and Application Thereof. CN109134492 (2019).

2. M. Zhang, J. Tamiya, L. Nguyen, M. W. Rowbottom, B. Dyck, T. D. Vickers, J. Grey, D. A. Schwarz, C. E. Heise, J. Haelewyn, M. S. Mistry, V. S. Goodfellow, Thienopyrimidinone Bis-Aminopyrrolidine Ureas as Potent Melanin-Concentrating Hormone Receptor-1 (MCH-R1) Antagonists. Bioorg. Med. Chem. Lett. 17, 2535–2539 (2007).

3. A. Cohen, P. Suzanne, J. C. Lancelot, P. Verhaeghe, A. Lesnard, L. Basmaciyan, S. Hutter, M. Laget, A. Dumètre, L. Paloque, E. Deharo, M. D. Crozet, P. Rathelot, P. Dallemagne, A. Lorthiois, C. Hopkins Sibley, P. Vanelle, A. Valentin, D. Mazier, S. Rault, N. Azas, Discovery of new thienopyrimidinone derivatives displaying antimalarial properties toward both erythrocytic and hepatic stages of Plasmodium. Eur. J. Med. Chem. 95, 16–28 (2015).
